# Sex differences in fear memory consolidation via Tac2 signaling in mice

**DOI:** 10.1038/s41467-021-22911-9

**Published:** 2021-05-03

**Authors:** A. Florido, E. R. Velasco, C. M. Soto-Faguás, A. Gomez-Gomez, L. Perez-Caballero, P. Molina, R. Nadal, O. J. Pozo, C. A. Saura, R. Andero

**Affiliations:** 1 Institut de Neurociències, Universitat Autònoma de Barcelona, Cerdanyola del Vallès, Barcelona, Spain; 2 Departament de Psicobiologia i de Metodologia de les Ciències de la Salut, Universitat Autònoma de Barcelona, Cerdanyola del Vallès, Barcelona, Spain; 3 Department de Bioquímica i Biologia Molecular, Facultat de Medicina, Universitat Autònoma de Barcelona, Cerdanyola del Vallès, Barcelona, Spain; 4 Centro de Investigación Biomédica en Red Enfermedades Neurodegenerativas (CIBERNED), Instituto de Salud Carlos III, Madrid, Spain; 5 Integrative Pharmacology and Systems Neuroscience Research Group, Neurosciences Research Program, IMIM (Hospital del Mar Medical Research Institute), Barcelona, Spain; 6 Unitat de Fisiologia Animal, Departament de Biologia Cel·lular, Fisiologia i Immunologia. Facultat de Biociències, Universitat Autònoma de Barcelona, Cerdanyola del Vallès, Barcelona, Spain; 7 Centro de Investigación Biomédica En Red en Salud Mental (CIBERSAM), Instituto de Salud Carlos III, Madrid, Spain; 8 Unitat de Neurociència Traslacional, Parc Taulí Hospital Universitari, Institut d’Investigació i Innovació Parc Taulí (I3PT), Universitat Autònoma de Barcelona, Cerdanyola del Vallès, Barcelona, Spain; 9 ICREA, Pg. Lluís Companys 23, Barcelona, Spain

**Keywords:** Amygdala, Classical conditioning, Fear conditioning, Preclinical research

## Abstract

Memory formation is key for brain functioning. Uncovering the memory mechanisms is helping us to better understand neural processes in health and disease. Moreover, more specific treatments for fear-related disorders such as posttraumatic stress disorder and phobias may help to decrease their negative impact on mental health. In this line, the Tachykinin 2 (Tac2) pathway in the central amygdala (CeA) has been shown to be sufficient and necessary for the modulation of fear memory consolidation. CeA-Tac2 antagonism and its pharmacogenetic temporal inhibition impair fear memory in male mice. Surprisingly, we demonstrate here the opposite effect of Tac2 blockade on enhancing fear memory consolidation in females. Furthermore, we show that CeA-testosterone in males, CeA-estradiol in females and Akt/GSK3β/β-Catenin signaling both mediate the opposite-sex differential Tac2 pathway regulation of fear memory.

## Abstract (web summary)

The Tachykinin 2 (Tac2) pathway in the central amygdala is sufficient and necessary for modulating fear memory consolidation. The authors show that silencing Tac2 neurons in the amygdala of male mice reduces fear expression, while fear expression in female mice is increased when manipulations are made during proestrus.

## Introduction

Memory is the process by which knowledge of the world is encoded, stored, and later retrieved by neural circuits^1^. As a widely accepted valid model for memory, the process of lasting memory formation is known to involve two different stages: short-term memory (which lasts min to hours) and long-term memory (which can last days, weeks, or even years). A major difference between them is that long-term memory requires a consolidation process in which gene regulation and protein synthesis are necessary. Fear memory is related to aversive events and allows an organism to identify threatening cues previously associated with a negative experience^2^. These cues evoke fear responses that are oriented to preserve the organism’s integrity in the face of danger, allowing the animals to reduce the probability of harm when the dangerous associated event appears. In the laboratory, we use Pavlovian fear conditioning to assess fear memory, which consists of pairing a neutral cue as a tone with an aversive event as a footshock, resulting in fear responses that may last for days when the conditioned cue is presented. Several data indicate notable differences in fear memory processing between the female and male brain, in both rodents and humans, at molecular, systemic, and behavioral levels of analysis^3,4^. Some of these differences include anatomical differences in the amygdala of rodents, as larger and denser medial amygdalae in males, but without differences in basolateral or central amygdala^5–7^. Unfortunately, the study of sex differences is still not fully considered in the scientific community researching the brain—including memory—although women are more likely to present a fear-related disorder^4^. In the last years, researchers have published 5.5 studies in males per 1 in females^8^ pointing out the evident and growing need to change our approach to neuroscience by including female subjects at all levels of research^9^. Consequently, the molecular mechanisms underlying sex differences between males and females on memory processing are still largely unclear.

**Fig. 1:**
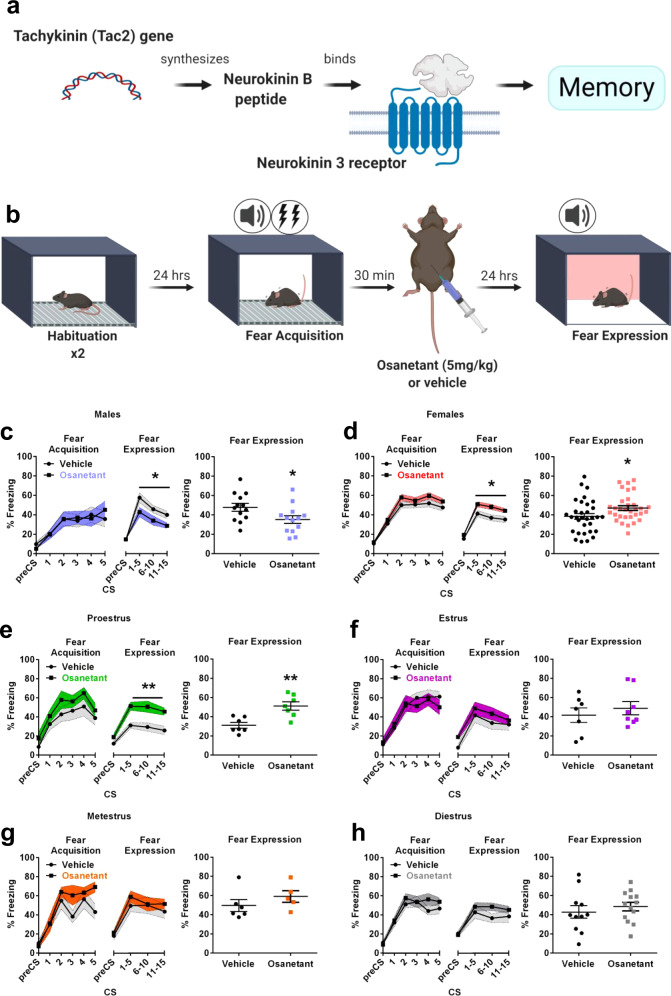
Systemic injection of osanetant given after 30 min of fear acquisition impairs memory consolidation in adult males and enhances it in females, especially during proestrus. **a** Schematic representation of the Tac2 pathway. **b** Experimental assessment of fear memory consolidation procedure. **c** Effect in the fear memory test 24 h after administration of osanetant (5 mg/Kg, ip) or vehicle 30 min after fear acquisition (FA) in adult males (*n* = 13 per group) (*p* = 0.036). **d** Effect in the fear memory test 24 h after administration of osanetant (5 mg/kg, ip) or vehicle 30 min after FA in adult females (*n* = 32 per group) (*p* = 0.014). This experiment was replicated three times with similar results (see Source Data file). Main effect treatment, main effect CS, main effect Sex and interactions were analyzed using two-way repeated measures ANOVA. **e** Effect in the fear memory test 24 h after administration of osanetant (5 mg/Kg, ip) or vehicle 30 min after FA in adult females divided by their estrous cycle on the FA and drug administration day in proestrus (*n* = 7 per group) (*p* = 0.004), (**f**) estrus (vehicle: *n* = 7; osanetant: *n* = 8), (**g**) metestrus (vehicle: *n* = 5; osanetant: *n* = 6) or (**h**) diestrus (vehicle: *n* = 11; osanetant: *n* = 13). The effect of the treatment for each estrous cycle stage was analyzed using one-way repeated measures ANOVA. Data are expressed as mean ± SEM. *p ≤ 0.05 **p ≤ 0.01 vs. vehicle. Asterisks indicate a statistically significant main effect treatment in repeated measures ANOVA.

We previously reported that the Tachykinin 2 (Tac2) pathway is sufficient and necessary for the modulation of fear memories^10^. The Tac2 gene encodes the neuropeptide Neurokinin B (NkB) that binds to the Neurokinin 3 receptor (Nk3R) (Fig. 1a). Nk3R couples to the pertussis toxin-insensitive G protein Gq, whose activation results in the production of inositol triphosphate and diacylglycerol, and the subsequent activation of protein kinase C resulting in memory modulation^10,11^. Tachykinins are abundant peptides in the central nervous system and are involved in neurotransmission and neuromodulation^12^. Because of its restricted brain expression in brain areas related to fear, such as the Bed Nucleus of the Stria Terminalis (BNST) or the centromedial amygdala (CeM) and the available tools, Tac2 manipulation allows a high degree of precision for modulating specific fear memory circuits^10^. The use of Tac2-Cre mice and Cre-dependent viral vectors for specific manipulations of these neurons allows the experimenter to manipulate a small population of neurons without affecting surrounding populations. Moreover, Nk3R antagonists, such as osanetant, are safe and well-tolerated drugs in humans meaning that these findings could rapidly be translated into humans^12^. Interestingly, Nk3R agonists or antagonists are not approved for the treatment of human disorders yet. It is known that there are differences across species in the distribution and pharmacology of the Nk3R in humans and rats^13^, but both species have a similar expression of Nk3R in the amygdala, an important area for fear memory regulation^13–15^. Furthermore, the binding affinity for NkB in rats and humans is equivalent^15^ suggesting similarities in the pharmacological properties of the Nk3R in both species.

Men and women have previously demonstrated differential life prevalence of fear-related disorders^16^. Specifically, females present a higher lifetime prevalence of fear-related disorders as posttraumatic stress disorder (PTSD) or specific phobias. Notwithstanding, the studies of the Tac2 pathway have been mostly performed only with male subjects. Here, we have discovered opposite-sex effects mediated by the Tac2 pathway in behavioral and molecular mechanisms of long-term memory in mice.

In this study, we explored the role of Tac2 signaling pathway on fear memory consolidation in both male and female young and adult mice. Importantly, we showed that both the pharmacological blockade of NK3R and chemogenetic silencing of Tac2 neurons in the CeM of male mice leads to subsequently reduced fear expression, while in females it increases the expression of fear when manipulations are made during proestrus. We also described the Akt/GSK3β/β-Catenin as the molecular pathway involved in the above-mentioned effects through its interaction with sex hormones in both, males and females.

## Results

### Nk3R antagonism decreases memory consolidation in adult males and increases it in adult female mice

Systemic administration of the Nk3R antagonist osanetant (5 mg/Kg) has previously shown to decrease fear memory consolidation in adult male mice when administered after fear acquisition (FA) by using a cued-fear conditioning (FC) paradigm (Fig. 1a, b)^10^. First, we hypothesized a similar regulation of fear memory consolidation in female mice than in males in a dose-dependent manner.

Surprisingly, 24 h after the administration of osanetant, female mice presented higher rates of freezing than the control group, thus showing increased memory consolidation in a totally opposite effect than in male mice, which showed decreased consolidation as previously reported^10^ (Sex x Treatment interaction: *F*_(1,86)_ = 10.087, *p* = 0.002; males: Fig. 1c, *F*_(1,24)_ = 4.964, *p* = 0.036; females: Fig. 1d, *F*_(1,62)_ = 6.133, *p* = 0.016). Another study reflects the fact that female mice may present different fear behaviors that may be of interest in the form of active fear responses instead of passive, such as darting^17^. Thus, we also studied darting behavior in male and female mice, although we found no significant differences between sexes (*F*_(1,11)_ = 0.672, *p* = 0.430). Further, female and male mice did not exhibit conditioned darting responses (Supplementary Fig. 1a, *F*_(1,1.997)_ = 1.086, *p* = 0.079).

Moreover, because it has already been proven that Nk3R systemic antagonism in humans decreases the level of circulating sex hormones^18^, and these have shown to be important for fear memory consolidation in rodents, we next tested whether the effect of osanetant (5 mg/Kg) was modulated by the estrous cycle in female mice or the dominance status, as a predictor for circulating testosterone concentration in both sexes. First, we explored whether an intrinsic variable among females could be modulating the effect of the drug. Based on previous works by Milad^19,20^ showing that the estrous cycle modulates the consolidation of fear extinction memories, we divided the animals of the previous experiment according to their estrous cycle stage on the day of FA and drug administration using the vaginal smear cytology method for the estrous cycle determination^21^. We found that osanetant is effective for increasing fear memory consolidation when FA and drug administration happen during proestrus (Fig. 1e, *F*_(1,12)_ = 12.590, *p* = 0.004)—presenting high concentrations of estradiol and progesterone in comparison to the rest of the cycle—but lack any effect during estrus (Fig. 1f, *F*_(1,13)_ = 0.492, *p* = 0.495), metestrus (Fig. 1g, *F*_(1,9)_ = 0.376, *p* = 0.555) or diestrus (Fig. 1h, *F*_(1,22)_ = 1.446, *p* = 0.242).

Besides, in males, to assess the interaction of sex hormones with the effect of osanetant as we did with females, we used the Confrontation Tube Test (CTT) to investigate whether social hierarchy could be influencing the effect of the drug on fear memory consolidation as suggested^9^. Because differential levels of circulating sex hormones appear to be key mediating the effect of osanetant on fear memory consolidation in female mice, we next tested whether differential concentrations of circulating testosterone also modulate the effect of the drug in male and female mice. The CTT is a tool that may be used to infer the dominance status of an animal and thus, differential testosterone levels in grouped-housed mice (Supplementary Fig. 2a)^9,22,23^. We found that both dominant and submissive male and female mice presented the same amount of fear expression 24 h after the administration of osanetant (males: Supplementary Fig. 2b, c, *F*_(1,12)_ = 0.226, *p* = 0.643; proestrous females: Supplementary Fig. 2d, e, *F*_(1,14)_ = 0.705, *p* = 0.415), showing no effect of social hierarchy in the effects of osanetant modulating fear memory consolidation. Males did not present differences in regard to social hierarchy in circulating corticosterone in relatively basal conditions (*F*_(1,13)_ = 0.423, *p* = 0.528), before habituation to CTT (*U* = 20, *p* = 0.565), before CTT (*F*_(1,13)_ = 0.011, *p* = 0.919), before habituation to FA chamber (*F*_(1,13)_ = 0.760, *p* = 0.400), before FA (*F*_(1,13)_ = 0.260, *p* = 0.620) or before FE test (*U* = 23, *p* = 0.848) (Supplementary Fig. 2f). In contrast, submissive females showed increased serum corticosterone in relatively basal conditions (*F*_(1,15)_ = 6.372, *p* = 0.024), before habituation to CTT (*F*_(1,15)_ = 35.715, *p* < 0.000), before CTT (*F*_(1,15)_ = 22.729, *p* < 0.000) and before habituation to FA chamber (*F*_(1,15)_ = 54.978, *p* < 0.000); but not before FA (*F*_(1,15)_ = 0.358, *p* = 0.559) or before FE test (*F*_(1,15)_ = 0.353, *p* = 0.562) (Supplementary Fig. 2g). Because the hierarchical status remained quite stable along time in females, we discarded the role of the estrous cycle mediating social dominance in adult females.

After demonstrating that the 5 mg/Kg dose in proestrous females and males, independent of social hierarchy, was modulating fear memory consolidation, we explored whether other doses had similar effects. For this, we tested both 1 mg/Kg and 10 mg/Kg finding that none of them altered fear memory consolidation in males (*F*_(1,21)_ = 0.876, *p* = 0.431), females during proestrus (*F*_(1,20)_ = 0,155, *p* = 0.858) or females during metestrus (*F*_(1,21)_ = 0.01, *p *= 0.990) (Supplementary Fig. 1b–d). Thus, the drug describes an inverted U shape dose–response curve which is similar to other drugs modulating memory^24^.

**Fig. 2:**
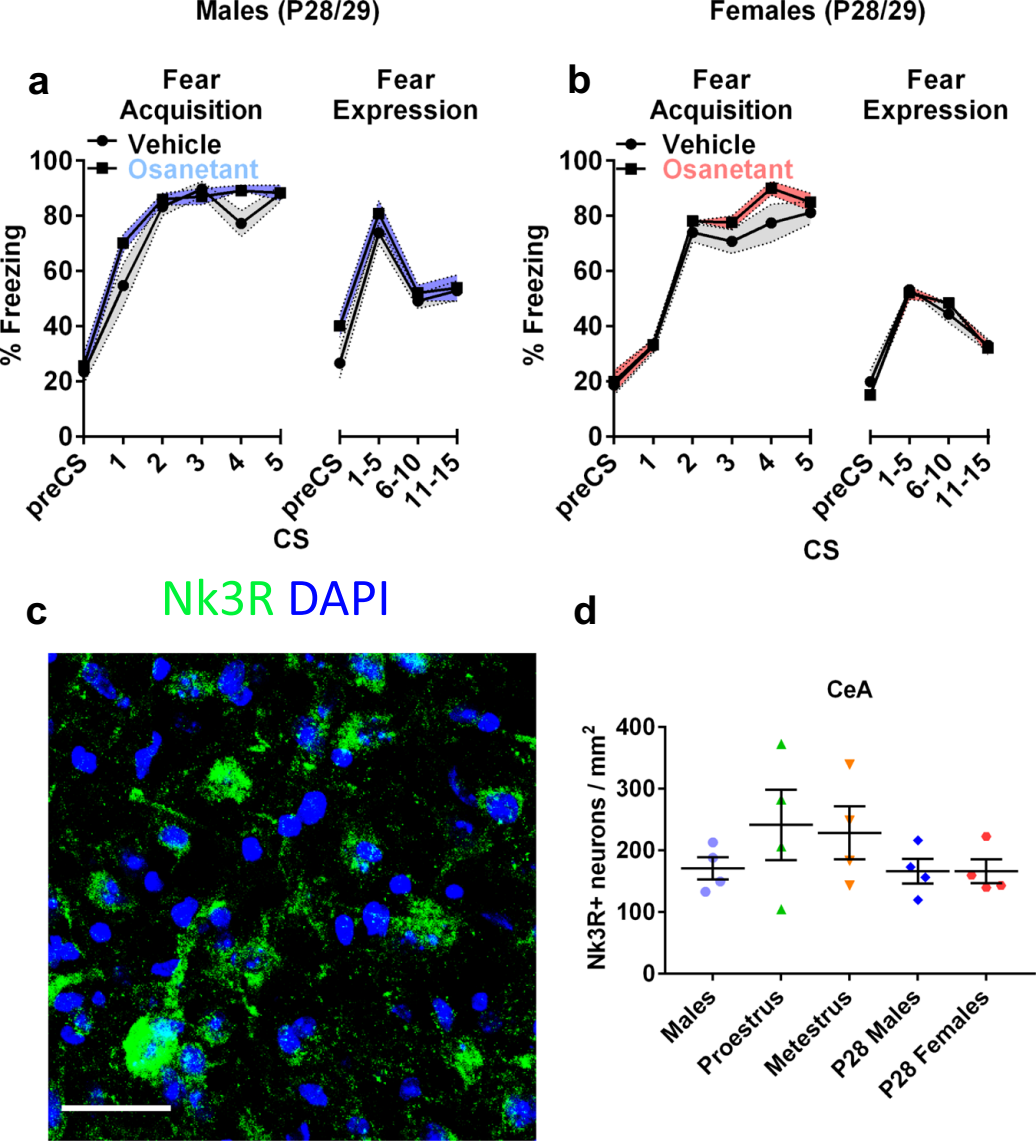
Systemic injection of osanetant given 30 min after fear acquisition is ineffective in modulating fear memory consolidation in prepubertal male and female mice, which present similar expression of Nk3R-positive neurons compared to adult male and female mice across the estrous cycle. **a** Effect of osanetant or vehicle administration 30 min after FA in freezing behavior in P28 male (*n* = 8 per group) and (**b**) female mice (*n* = 8 per group). **c** Representative confocal image of the Nk3R in the CeA of prepubertal mice. Scale bar = 60 µm. **d** Quantification of Nk3R-positive cells in the CeA of adult and prepubertal males and prepubertal and adult females across the cycle (*n* = 4 per group). Data are expressed as mean ± SEM. Main effect treatment, main effect CS, and CSxTreatment interaction were analyzed using repeated measures ANOVA, while NK3R expression was analyzed using non-parametric Krukal–Wallis’ χ^2^.

Due to the suggested role of sex hormones in modulating the effect of osanetant on fear memory consolidation, we wanted to test whether this modulation was dependent on the maturation of the organism at the moment of receiving the drug, because of the differences involved in circulating sex hormones and the maturation of the brain systems involved. To that end, we tested 5 mg/Kg of osanetant on prepubertal postnatal day (P) P28 in male and female mice, which have not reached sexual maturity yet^25^. Results showed that male and female mice at day P29 had similar freezing rates irrespective of treatment (males: Fig. 2a, *F*_(1,14)_ = 0.171, *p* = 0.686; females: Fig. 2b, (*F*_(1,14)_ = 0.037, *p* = 0.851), thereby suggesting that osanetant does not modulate fear memory consolidation in the absence of adult levels of sex hormones in males. An alternative explanation may be that the Tac2 pathway circuits are not mature yet although the neuropeptide is expressed in the brain. The lack of effect in prepubertal mice might not only be due to the lack of adult levels of sex hormones, but developmental factors may interfere such as the maturation of the fear-related neurocircuitry. Further, both young and adult females and males, express similar levels of Nk3R in the central amygdala (CeA) (Fig. 2c, d, χ^2^_(3)_ = 2.991, *p* = 0.393).

Our results showed a completely opposite-sex effect of the drug in male and female mice, with males presenting a reduction of fear memory consolidation and females an increase after receiving osanetant. Further, in females, the effect of the drug was clearly visible when administered during the proestrous stage of the estrous cycle, but not during other stages. Osanetant was effective in modulating fear memory consolidation at 5 mg/Kg, but not at higher (10 mg/Kg) or lower (1 mg/Kg) doses. Moreover, social hierarchy, as a predictor of circulating testosterone, did not show to modulate the effect of osanetant on fear memory consolidation in males or females. Importantly, P28 male and female mice that received the drug did not present an effect on fear memory consolidation, highlighting the importance of sexual maturity for osanetant to modulate memory consolidation in both sexes.

### Osanetant decreases males’ testosterone and increases females’ estradiol during the consolidation window of fear memories

Taking into account previous reports that show a decrease of circulating sex hormones in humans due to the administration of an Nk3R antagonist^18^, we hypothesized that the observed behavioral effects were mediated by a similar decrease in circulating testosterone and estradiol, without altering stress hormones. Besides, because adult sex hormones appeared to be necessary for osanetant to modulate memory consolidation in males and females, we next tested the hormonal dynamics after FA and injection of osanetant or vehicle. To prove our hypothesis, we measured sex and stress hormones at two different time points (30 min and 330 min after the injection of osanetant) that reflected different moments of the consolidation window of fear memories.

**Fig. 3:**
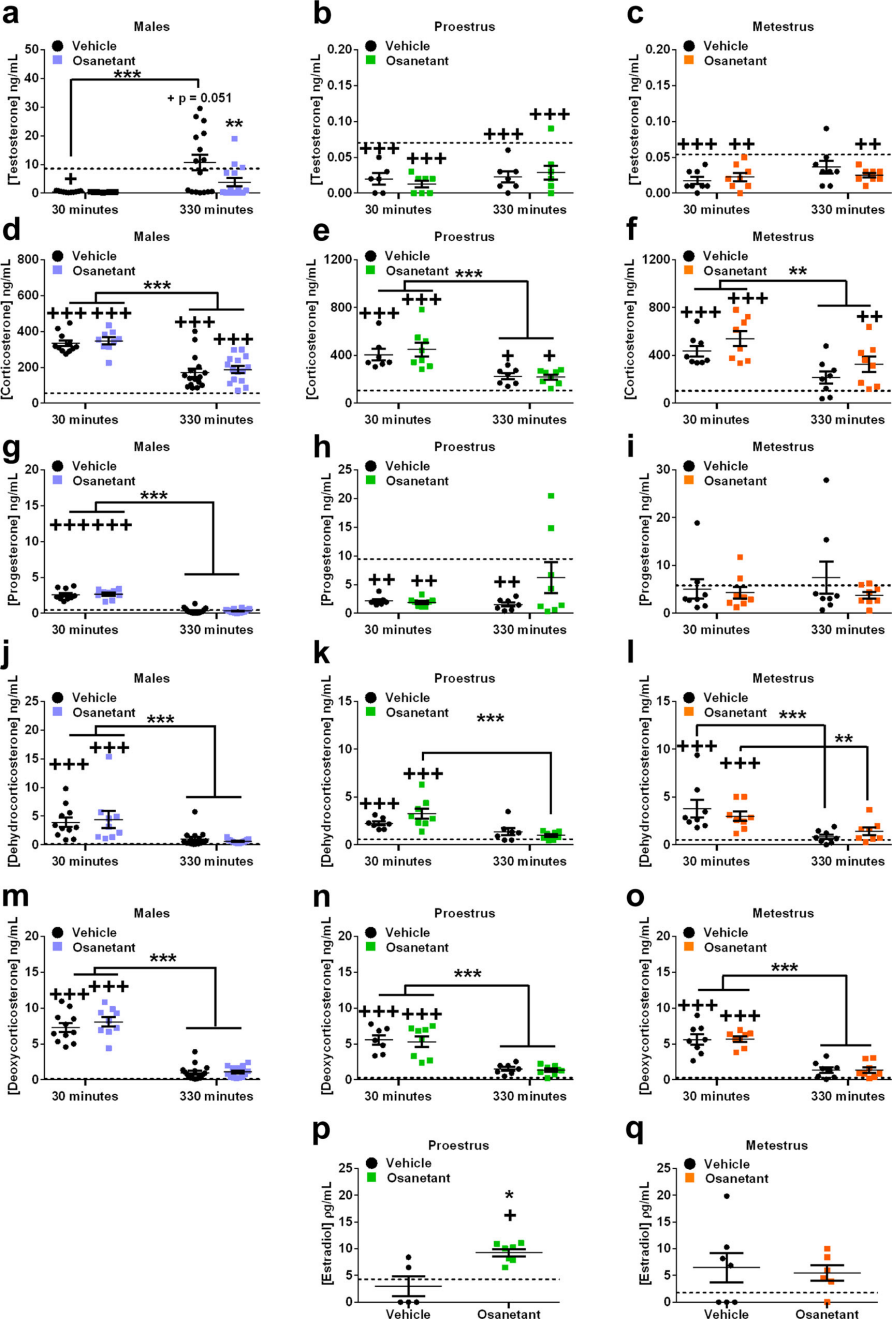
Testosterone levels in serum in males after fear acquisition are decreased by osanetant while estradiol in females’ serum is increased 330 min after its administration compared to vehicle-treated mice, with no effect of the drug on progesterone, corticosterone, deoxycorticosterone, or dehydrocorticosterone. All mice received osanetant (5 mg/Kg, ip) or vehicle 30 min after fear acquisition, and trunk blood was collected either 30 min or 330 min after the injection. Estradiol measures were only determined 330 min after the injection. Dashed lines indicate the relatively basal concentration for an independent group of animals that did not receive any treatment. **a**–**c** Testosterone concentration in males (*p* = 0.001, treatment_330_min: *p* = 0.007), proestrous females, and metestrous females. **d**–**f** Serum corticosterone concentration in males (p = 0.000), proestrous females (*p* = 0.000), and metestrous females (*p* < 0.000). **g**–**i** Circulating progesterone concentration in serum in males (*p* = 0.000), proestrous females, and metestrous females. **j**–**l** Males (*p* < 0.000), proestrous (*p* < 0.000), and metestrous (*p* < 0.000) females’ serum dehydrocorticosterone (DHC) concentration. **m**–**o** Concentration of circulating deoxycorticosterone (DOC) in serum in males (*p* = 0.000), proestrous (*p* < 0.000) and metestrous females (*p* < 0.000). **p** Serum estradiol concentration after FA and 330 min after receiving osanetant in proestrous females (*p* = 0.022). **q** Serum estradiol concentration after FA and 330 min after receiving osanetant in metestrous females. Basal levels of the estradiol experiment were replicated in Supplementary Fig. 3e with similar results. Males vehicle_30_: *n* = 11; males osanetant_30_: *n* = 10; males vehicle_330_: *n* = 16; males osanetant_330_: *n* = 16; proestrous females vehicle_30_: n = 8; proestrous females osanetant30: *n* = 8; proestrous females vehicle_330_: *n* = 7; proestrous females osanetant_330_: *n* = 8; metestrous females vehicle_30_: *n* = 8; metestrous females osanetant_30_: *n* = 8; metestrous females vehicle_330_: *n* = 8; metestrous females osanetant_330_: *n* = 8. Proestrous females vehicle_330_ for estradiol determination *n* = 5; proestrous females osanetant_330_ for estradiol determination *n* = 7; metestrous females vehicle_330_ for estradiol determination *n* = 7, metestrous females osanetant_330_ for estradiol determination *n* = 6. Data are mean ± SEM. *p ≤ 0.05 **p ≤ 0.01 ***p ≤ 0.001 vs its vehicle. When with lines, it indicates specific comparisons. ^+^*p* ≤ 0.05 ^++^*p* ≤ 0.01 ^+++^*p* ≤ 0.001 vs baseline. The statistic was Wald’s χ^2^ with pairwise comparisons between groups for testosterone, corticosterone, progesterone, DHC, and DOC. Estradiol was analyzed using non-parametric Kruskal–Wallis’ χ^2^.

Testosterone was reduced 30 min after the injection of the drug (1 h after FA) in males (χ^2^_(4)_ = 19.661, *p* = 0.001; vehicle_30_ vs. baseline *p* = 0.043, osanetant_30_ vs. baseline *p* = 0.050), females in proestrus (χ^2^_(4)_ = 31.645, *p* < 0.000; vehicle_30_ vs. baseline *p* < 0.000, osanetant_30_ vs. baseline *p* < 0.000) and females in metestrus (χ^2^_(4)_ = 17.296, *p* = 0.002; vehicle_30_ vs. baseline *p* < 0.000, osanetant_30_ vs. baseline *p* = 0.001) (Fig. 3a–c). When assessed 330 min after receiving osanetant or vehicle (6 h after FA), males that received vehicle presented a peak of testosterone above the baseline (vehicle_330_ vs. baseline *p* = 0.051) while the osanetant treated group presented lower levels than the vehicle group (vehicle_330_ vs. osanetant_330_ *p* = 0.007) (Fig. 3a), thus showing that the injection reduced the testosterone levels during the consolidation window of fear memories. Similar results were obtained with amygdalae micropunches; osanetant decreased amygdalar concentration of testosterone compared to vehicle 330 min after the administration of osanetant (6 h after FA) (Supplementary Fig. 3a, *U* = 9, *p* = 0.016). Furthermore, linear regression analyses showed that testosterone in serum was highly predictive of amygdalae testosterone (Supplementary Fig. 3c, *R*^2^ = 0.786, *p* < 0.001) as well as corticosterone (Supplementary Fig. 3d, *R*^2^ = 0.344, *p* = 0.017). In contrast, neither females presented an effect of the drug in testosterone when compared to the vehicle groups during proestrus (vehicle_30_ vs. osanetant_30_ *p* = 0.707, vehicle_330_ vs. osanetant_330_ *p* = 0.520) nor metestrus (vehicle_30_ vs. osanetant_30_ *p* = 0.686, vehicle_330_ vs. osanetant_330_ *p* = 0.283) (Fig. 3b, c). Females during proestrus presented increased estradiol concentration in serum compared to the vehicle and baseline 330 min after receiving the drug (Fig. 3p, χ^2^_(2)_ = 8.307, *p* = 0.016; osanetant_330_ vs. baseline *p* = 0.022, vehicle_330_ vs. osanetant_330_ *p* = 0.022), showing increased estradiol during the consolidation window of memory, while females during metestrus presented no effect of the drug (Fig. 3q, χ^2^_(2)_ = 2.678, *p* = 0.262).

We also tested whether osanetant produced an effect over stress-related hormones corticosterone, dehydrocorticosterone, or deoxycorticosterone, as well as in progesterone in males, proestrous and metestrous females. None of these hormones were modulated by the administration of osanetant in any of the two chosen time points (30 or 330 min after the drug injection) (Fig. 3d–o). This lack of modulation was also found in amygdala micropunches for corticosterone (Supplementary Fig. 3b, *t*_(14)_ = 1.263, *p* = 0.227). We found increased corticosterone in serum in response to the stress induced by FA 30 min after the treatments in males (χ^2^_(4)_ = 193.334, *p* < 0.000, vehicle_30_ vs. baseline *p* < 0.000, osanetant_30_ vs. baseline *p* < 0.000), females during proestrus (χ^2^_(4)_ = 62.980, *p* < 0.000, vehicle_30_ vs. baseline *p* < 0.000, osanetant_30_ vs. baseline *p* < 0.000) and females during metestrus (χ^2^_(4)_ = 52.572, *p* < 0.000, vehicle_30_ vs. baseline *p* < 0.000, osanetant_30_ vs. baseline *p* < 0.000). This increase in serum corticosterone was found to decrease over time between the two time points (males: vehicle_30_ vs. vehicle_330_ *p* < 0.000, osanetant_30_ vs. osanetant_330_ *p* < 0.000; females in proestrus: vehicle_30_ vs. vehicle_330_ *p* = 0.001, osanetant_30_ vs. osanetant_330_ *p* < 0.000; females in metestrus: vehicle_30_ vs. vehicle_330_ *p* = 0.001, osanetant_30_ vs. osanetant_330_ *p* = 0.001) (Fig. 3d–f). Dehydrocorticosterone and deoxycorticosterone followed similar patterns as corticosterone as expected (Fig. 3j–o).

Progesterone was increased independently of the treatment 30 min after receiving the injection in males (χ^2^_(4)_ = 464.285, *p* < 0.000, vehicle30 vs. baseline *p* < 0.000, osanetant_30_ vs. baseline *p* < 0.000) and decreased in proestrous females (χ^2^_(4)_ = 14.490, *p* = 0.006, vehicle_30_ vs. baseline *p* = 0.005, osanetant_30_ vs. baseline *p* = 0.003), but no changes were observed in females during metestrus (χ^2^_(4)_ = 2.136, *p* = 0.711). Males returned to relatively basal progesterone levels at 330 min after the injection (vehicle_330_ vs. baseline *p* = 0.114, osanetant_330_ vs. baseline *p* = 0.072), while in proestrous females only osanetant-treated mice recovered the baseline 330 min after the injection (vehicle_330_ vs. basal *p* = 0.002, osanetant_330_ vs. basal *p* = 0.200) (Fig. 3g–i).

Interestingly, osanetant did not alter corticosterone or related metabolites in any of the chosen time points in both males and females. Importantly for our study, osanetant-treated males presented lower testosterone than the ones administered with the vehicle at 330 min after receiving osanetant (360 min after FA). In osanetant-treated females, we also found an increase of estradiol in proestrus, but not during metestrus. Altogether, osanetant showed a sex-opposite regulation of sex hormones 330 min after its administration in male and female mice that underwent FA, in a similar direction than the behavioral effects on memory consolidation.

### Nk3R expression is similar in males and proestrous and metestrous females, with increased colocalization of Nk3R with GAD65 during proestrus

Nk3R is highly expressed under basal conditions in limbic and prefrontal cortical areas. Especially in the amygdala, it is highly localized in the CeA^26^. We have previously shown that the modulation of Nk3R in the CeA is crucial for the regulation of memory consolidation^10^. Thus, we hypothesized that osanetant was blocking Nk3R-expressing neurons in the CeA that were differentially colocalized with inhibitory biomarkers in males and excitatory biomarkers in females, which could be modulated by the estrous cycle. To test our hypothesis, we performed an immunohistochemistry assay in the CeA to measure colocalization of Nk3R with GAD65, CaMKIIα, or vGLUT2. Further, we compared CeA Nk3R-positive neurons between males, females in proestrus or metestrus and P28 male and female mice to assure the lack of effect in prepubertal mice is not due to a lack of expression of this receptor in CeA.

First, we found non-significant differences in Nk3R-positive neurons in the CeA in males, females in both proestrus and metestrus and prepubertal male and female mice (Fig. 2c, d, χ^2^_(3)_ = 2.991, *p* = 0.393). To explore for possible changes of neuronal markers in Nk3R-positive neurons in the CeA between males and females across the estrous cycle, which could explain the sex-dependent opposite effects on memory consolidation, we next examined the colocalization of Nk3R with markers of inhibitory neurons (GAD65), glutamate synaptic plasticity (CAMKIIα) and excitatory neurons (vGLUT2) (Supplementary Fig. 4c). Remarkably, colocalization of GAD65 with Nk3R was increased in females during proestrus when compared to metestrous females and males (Supplementary Fig. 4c, e, *F*_(2,10)_ = 6.001, *p* = 0.022, males vs. proestrus *p* = 0.015, metestrus vs. proestrus *p* = 0.015). Furthermore, CaMKIIα immunocolocalization with Nk3R was also increased in females in comparison to males (Supplementary Fig. 4c, e, *F*_(2,10)_ = 4.856, *p* = 0.037, males vs. proestrus *p* = 0.022, males vs. metestrus *p* = 0.027). Immunocolocalization of vGLUT2 with Nk3R showed no differences among groups (Supplementary Fig. 4c, e, *F*_(2,10)_ = 2.07, *p* = 0.189).

Given the evident role of estradiol in the effects of the drug on memory consolidation, an immunolocalization study was performed to assess colocalization of Estrogen Receptors (ERs: ERβ, and ERα) with Nk3R in the CeA. We found a significant sex effect in colocalization of ERβ with Nk3R, showing decreased colocalization of these receptors in the CeA in females compared to males (Supplementary Fig. 4a, d, *F*_(1,10)_ = 7.251 *p* = 0.023). On the other hand, we found no differences in colocalization of ERα with Nk3R between males and proestrous or metestrous females (Supplementary Fig. 4b, d, χ^2^_(2)_ = 1.423, *p* = 0.491).

Our results showed a similar expression of Nk3R in the CeA in adult and prepubertal male and female mice, independently of the estrous cycle. Interestingly, female mice showed increased colocalization of CaMKIIα with Nk3R compared to males, suggesting a higher tendency towards CaMKIIα-mediated synaptic plasticity in females. Interestingly, although we found no differences in colocalization of an excitatory biomarker, as vGLUT2, with Nk3R in the CeA; we found increased colocalization of GAD65 with Nk3R in the CeA of proestrous females, suggesting increased susceptibility to GABA-mediated synaptic plasticity in the CeA of females during proestrus.

### Chemogenetic silencing of CeA-Tac2 neurons after FA decreases memory consolidation in males and increases it in proestrous females

Because it has been previously shown that local Nk3R antagonism in the CeA decreases fear memory consolidation in male mice^10^, we next hypothesized that a single micro-infusion of osanetant in the CeA of females mice during proestrus would increase fear memory consolidation in a similar manner than the intraperitoneal injection. Further, due to the high degree of colocalization between NkB (the result of the *Tac2* gene expression) and Nk3R in the CeM^27^, we hypothesized that the silencing of Tac2 neurons in the CeM could replicate the osanetant-induced alterations on fear memory consolidation in male and female mice. Because Nk3R is also expressed in other areas of the amygdala where Tac2 is not synthesizing NkB, such as more lateral portions of the CeA^27^, we wanted to test whether the effect on memory consolidation was replicated by specific Tac2-CeM blockade.

**Fig. 4:**
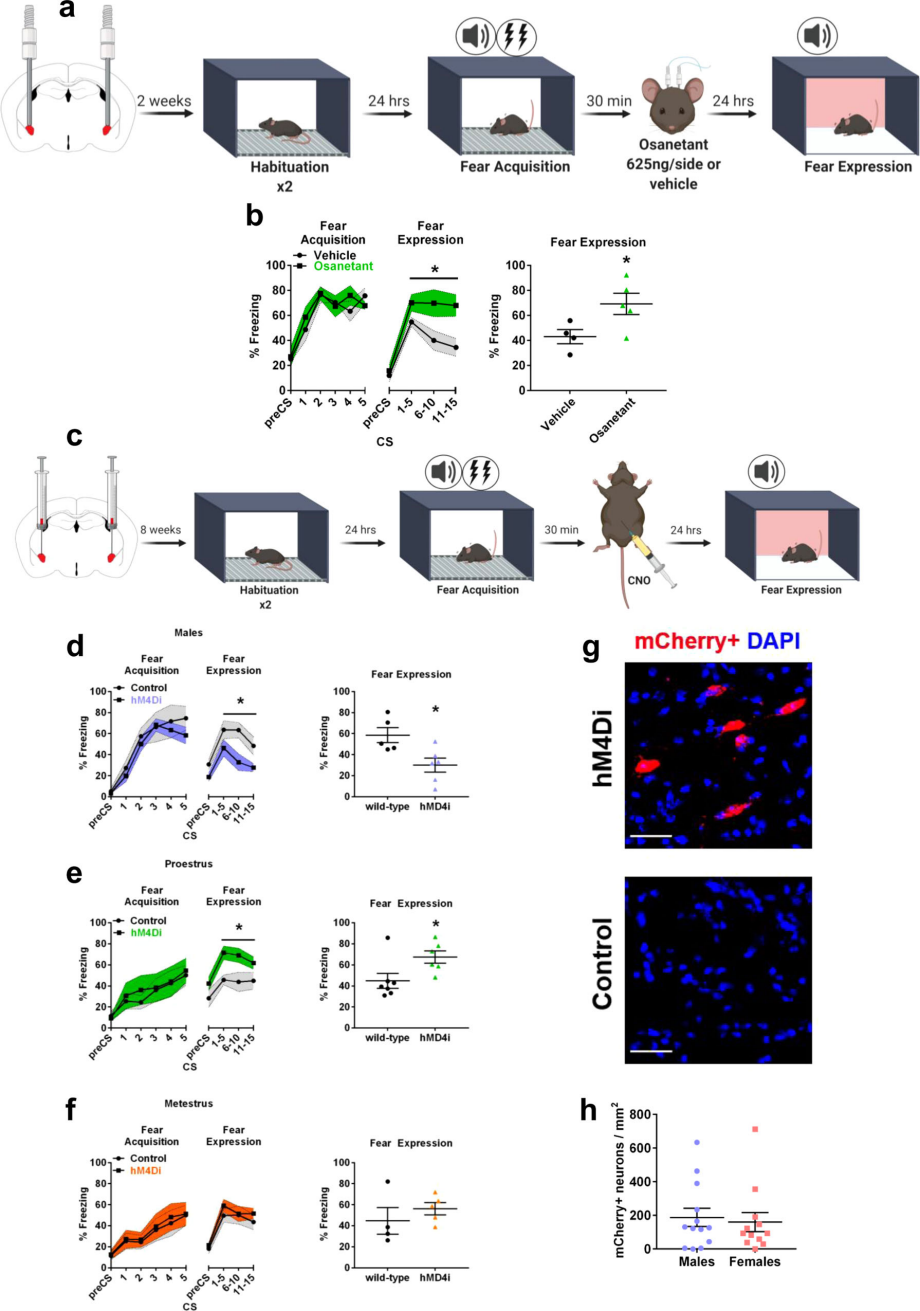
CeA-Nk3R blockade increases fear memory consolidation in proestrous females while chemogenetic silencing of the CeA-Tac2 neurons impairs fear memory consolidation in males and enhances it in females during proestrus. **a** Schematic diagram of intracerebral injection procedure with FC. **b** Effect in the fear memory test 24 h after osanetant (625 ng/side) or vehicle intracerebral injections in the Central Amygdala (CeA) after fear acquisition (FA) in proestrous females (vehicle: *n* = 4; osanetant: *n* = 5) (*p* = 0.046). Main effect treatment, main effect CS, and CSxTreatment interaction were analyzed using one-way repeated measures ANOVA. **c** Schematic diagram of a fear procedure to assess Gi DREADD mediated silencing of CeA-Tac2 neurons. **d** Temporal silencing of CeA-Tac2 neurons after FA effect on fear memory consolidation in male mice as measured by the Fear Expression (FE) test 24 h later (wild-type: *n* = 5; hMD4i: *n* = 6) (*p* = 0.036). **e** Temporal silencing of CeA-Tac2 neurons after FA effect on fear memory consolidation measured by FE test 24 h later in proestrous females (wild-type: *n* = 6; hMD4i: *n* = 7) (*p* = 0.034). **f** Temporal silencing of CeA-Tac2 neurons after FA effect on memory fear consolidation in metestrous females (wild-type: *n* = 4; hMD4i: *n* = 5). Main effect treatment, main effect CS, main effect sex and interactions were analyzed using two-way repeated measures ANOVA. The effect of the treatment for each estrous cycle stage was analyzed using one-way repeated measures ANOVA. **g** Representative confocal image showing mCherry-positive neurons in infected Tac2-Cre mice (hM4Di) but not in wild-type (control). Scale bar = 30 µm. **h** Males and females count of CeA-Tac2 mCherry-positive neurons per mm^2^ (males: *n* = 13; females: *n* = 12). Data are mean ± SEM. *p ≤ 0.05 vs. vehicle or wild-type. Asterisks above a line indicate significant main effect treatment in repeated measures ANOVA. Mann–Whitney’s U was used for the histochemistry assay.

Bilateral local injection of osanetant (625 ng/side) in the CeA of proestrous females (Fig. 4a, Supplementary Fig. 7) after FA increased memory consolidation, as expected, as shown by increased freezing rates during the expression test when compared to vehicle controls (Fig. 4b, *F*_(2,7)_ = 5.858, *p* = 0.046). This finding demonstrated that a population of Nk3R-positive neurons located in the CeA is required for the modulation of memory consolidation in an opposite-sex manner.

Next, we used Tac2-Cre mice to assess whether chemogenetic silencing of these neurons in the CeA replicated the effects of pharmacological Nk3R blockade in fear memory consolidation in both sexes. Male and female mice were inoculated in the CeA with the Cre-dependent AAV-DIO-hMD4i-mCherry. Tac2 neurons were silenced with CNO administration (1 mg/Kg, ip) 30 min after FA (Fig. 4c)^10^. The fear expression test revealed reduced freezing in male mice that expressed the reporter mCherry in the CeA when compared to wild-type injected controls. In contrast, memory consolidation was significantly increased in female mice with expression of the reporter in the CeA (Sex x Genotype interaction: *F*_(1,31)_ = 1.972, *p* = 0.002; males: Fig. 4d, *F*_(1,9)_ = 5.738, *p* = 0.036; females: *F*_(1,20)_ = 5.618, *p* = 0.028), with different effects according to the estrous cycle on the FA and drug administration day. Females that expressed mCherry in the CeA, and received FA and CNO in proestrus presented increased memory consolidation (Fig. 4e, *F*_(1,11)_ = 5,897, *p* = 0.034), while this effect was absent during metestrus (Fig. 4f, *F*_(1,7)_ = 0.784, *p* = 0.405), all compared to wild-type controls also injected with the AAV without the reporter.

The expression of the reporter mCherry was also used to assess the number of Tac2-positive neurons in the CeA (Fig. 4g). Supporting our previous Nk3R-positive immunolocalization results, no difference was found in the number of Tac2-positive neurons between males and females in the CeA (Fig. 4h, *U* = 68, *p* = 0.586). These data indicated that the opposite-sex effect on memory consolidation was not due to a different number of CeA-Tac2-positive neurons.

Our results suggest that osanetant-mediated alterations on fear memory consolidation occur because of a blockade of Tac2 neurons in the CeM rather than because of possible peripheral effects caused by the drug. Moreover, we concluded that the sex-opposite effect observed was not due to a different expression of Tac2 in the CeM, as shown by similar expression of the reporter mCherry between sexes.

### Osanetant oppositely regulates the Akt/GSK3β/β-Catenin pathway in the amygdala during memory consolidation

Nk3R is a G Protein-Coupled Receptor (GPCR) upstream of different signaling pathways regulating the synthesis of inositol triphosphate^11^. To detect the downstream signaling pathways affected by the administration of osanetant 30 min after FA, we performed an exploratory experiment. Next, we confirmed our results using Western Blot and further identified a specific route altered in CeA cells in response to osanetant during the consolidation window of fear memories.

**Fig. 5:**
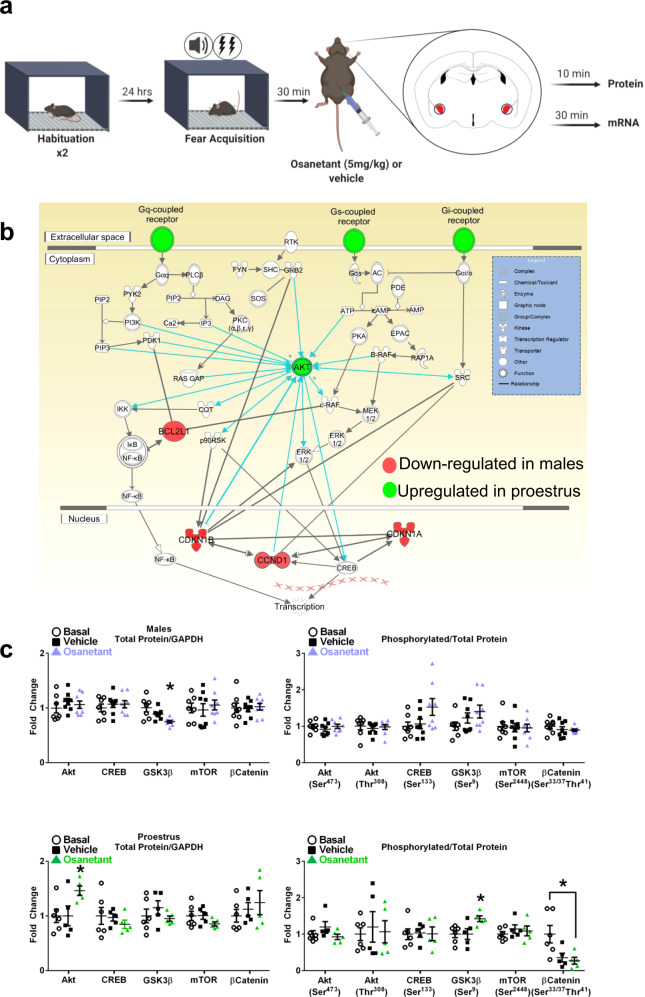
Osanetant administered 30 min after FA reduces Akt/GSK3β/β-Catenin pathway activation in males and it enhances it in proestrous females in the amygdala. **a** Fear procedure to obtain amygdala tissue for biochemical studies. **b** Schematic representation of the involved PI3K/Akt pathway revealed by the G-Coupled Protein Receptor (GCPR) qPCR array results after bioinformatics analysis of the Ingenuity Pathway Analysis (IPA) software. Red indicates downregulation and green upregulation (*n* = 4 per group). **c** Western blot analyses of the Akt/GSK3β/β-Catenin signaling pathway in amygdalar lysates of males in males (basal: *n* = 7; vehicle: *n* = 8; osanetant: *n* = 8) revealed downregulated total GSK3β expression in osanetant treated mice compared to vehicle (*p* = 0.006) and basal (*p* = 0.011) groups; while proestrous females (basal: *n* = 6; vehicle: *n* = 5; osanetant: *n* = 5) showed increased total Akt expression in osanetant treated mice compared to vehicle (*p* = 0.035) and basal groups (*p* = 0.029), with a decrease in p-β-Catenin in osanetant treated females compared to basal animals (*p* = 0.017). Original blots and scans are shown in Supplementary Fig. 6. Protein levels were normalized to GADPH (left graphs) and phosphorylated proteins were normalized to total protein levels (right graphs). Data are mean ± SEM. *p ≤ 0.05 vs. the other groups. For qPCR analysis, two-tailed t-test or Mann–Whitney’s U test were used for statistical analyses. Western blots were analyzed with one-way ANOVA or Kruskal–Wallis’ χ^2^ test. Pairwise comparisons are indicated when appropriate.

To that end, we dissected both amygdalae 30 min after receiving osanetant after fear acquisition in males and females during proestrus and metestrus (Fig. 5a). A qPCR mRNA array of 84 GPCR-related genes was performed, revealing downregulation of *Agt, Agtrap, Bcl2, Calcrl, Ccnd1, Ccne1, Cdkn1a, Cdkn1b, Cflar, Elk4, Galr2, Gnas, Bcl2ll* in males that received osanetant compared to vehicle controls; while *Akt1, Gcgr, Fgf2, Lhcgr, Mmp9, Ptgdr, Rho, S1pr3, Vcam1* gene expression was upregulated in females that received osanetant only during proestrus (Supplementary Fig. 5). In females during metestrus, only *Adrb2* and *Lpar1* were downregulated and *Kcnh8* upregulated after the treatment (Supplementary Table 1). Ingenuity Pathway Analysis (IPA) of these genes revealed the Akt/GSK3β/β-Catenin signaling pathway as the main molecular route affected by the drug, and specifically, Akt signaling was upregulated in proestrus (Fig. 5b, Supplementary Fig. 6).

Since PI3K/Akt signaling targets different pathways, we next analyzed the activation and protein levels of its three main downstream pathways—CREB, mTOR, and GSK3β—following a similar approach (Fig. 5a). 10 min after the injection of the drug, amygdalae were dissected and levels of total and phosphorylated levels of CREB, mTOR, GSK3β, and Akt were analyzed by Western blotting. Our results showed no effect of the drug in males or proestrous females in the levels of total CREB (males: *F*_(2,21)_ = 0.332, *p* = 0.722; proestrus: χ^2^_(3)_ = 0.876, *p* = 0.645), phosphorylated CREB (Ser 133) (males: χ^2^_(3)_ = 4.182, *p* = 0.124; proestrus: *F*_(2,14)_ = 0.014, *p* = 0.986), total mTOR (males: *F*_(2,21)_ = 0.210, *p* = 0.813; proestrus: *F*_(2,14)_ = 1.441, *p* = 0.272) or phosphorylated mTOR (Ser 2448) (males: *F*_(2,21)_ = 0.049, *p* = 0.952; proestrus: *F*_(2,14)_ = 1.441, *p* = 0.632) (Fig. 5c, Supplementary Fig. 6). Notwithstanding, total GSK3β protein levels were significantly decreased in males (Fig. 5c, Supplementary Fig. 6, χ^2^_(3)_ = 10,031, *p* = 0.007, basal vs. osanetant *p* = 0.011, vehicle vs osanetant *p* = 0.006) while in females during proestrus total Akt expression was increased (*F*_(2,14)_ = 3.821, *p* = 0.050, basal vs. osanetant *p* = 0.029, vehicle vs. osanetant *p* = 0.035), but not its phosphorylation (Ser 473 and Thr 308) (respectively: χ^2^_(3)_ = 3.640, *p* = 0.162; χ^2^_(3)_ = 0.051, *p* = 0.975). In agreement with elevated Akt, phosphorylated GSK3β (Ser 9)/GSK3β ratio was increased (*F*_(2,14)_ = 4.664, *p* = 0.030, basal vs. osanetant *p* = 0.017, vehicle vs. osanetant *p* = 0.022). Inactive phosphorylated GSK3β may lead to decreased phosphorylation of its downstream target β-Catenin (Ser33/37, Thr41) (χ^2^_(3)_ = 6887, *p* = 0.032; basal vs. vehicle *p* = 0.052, basal vs. osanetant *p* = 0.017, osanetant vs. vehicle *p* = 0.841) (Fig. 5c, Supplementary Fig. 6). These results are in agreement with the observed increase of Akt1 transcript levels in the gene expression analyses and indicate that osanetant-mediated Nk3R blockade results in upregulation of Akt leading to inactivation of GSK3β and upregulation of β-Catenin in proestrous females.

In summary, males and proestrous females presented a sex-opposite regulation of the PI3K/Akt pathway in the CeA after receiving osanetant as shown by bioinformatic analyses of the GPCR-related genes expression patterns. Further, Western Blots confirmed these results and revealed a downregulation of the Akt/GSK3β/β-Catenin in male mice while this downstream pathway was upregulated in female mice during proestrus, thus, suggesting to be mediating the observed effects on fear memory consolidation in both sexes.

### Blockade of osanetant effect on memory consolidation by androgen receptor (AR) agonism or Akt activation in males and ERs antagonist or Akt inactivation in females

Altogether, osanetant reduced testosterone in males and increased estradiol in proestrous females during memory consolidation. Further, we also found that osanetant decreased the Akt/GSK3β/β-Catenin pathway in males while it increased it in proestrous females. Therefore, we hypothesized that local activation in the CeA of AR in males and blockade of ERs in proestrous females would revert the consolidation effects caused by osanetant. Moreover, our hypothesis also contemplated that local activation of Akt in males, and inhibition in females would also revert the mentioned effects of osanetant.

**Fig. 6:**
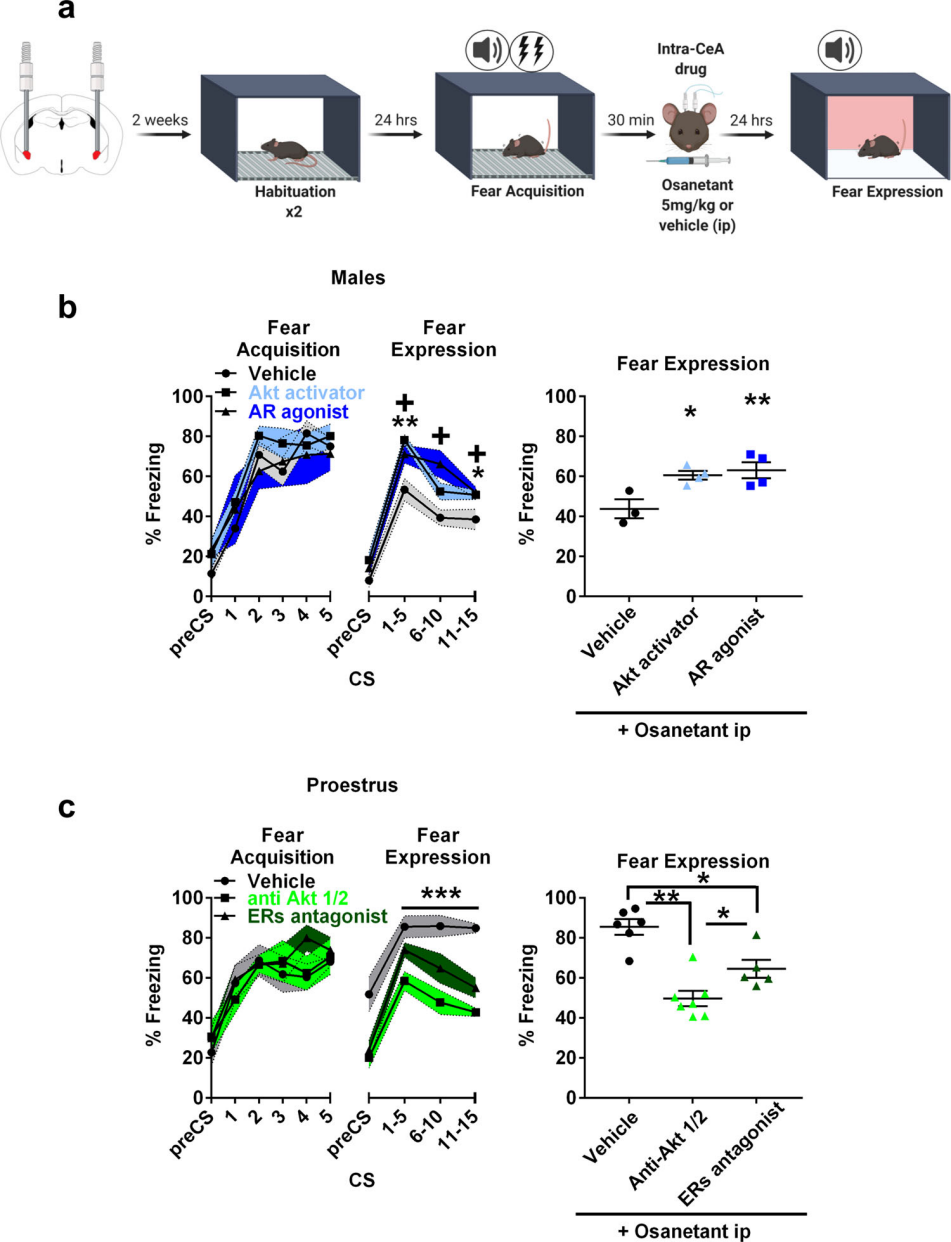
Downregulation of CeA-Akt pathway and testosterone in males and upregulation of CeA-Akt signaling and estradiol in proestrous females are necessary for the systemic effect of osanetant in memory. **a** Schematic procedure for intra-CeA infusion of drugs after Fear Acquisition (FA). All mice received osanetant ip. Males received intra-CeA Akt activator, Androgen Receptor (AR) agonist or vehicle. Females received intra-CeA Akt inhibitor, Estrogen Receptors (ERs) antagonist or vehicle. **b** Effect of an acute administration of an Akt activator or an AR agonist in osanetant treated adult males on fear memory consolidation (vehicle *n* = 3; Akt activator *n* = 4; androgen receptor agonist *n* = 4) (against vehicle: Akt activator *p* = 0.013; AR agonist: *p *= 0.006). *p ≤ 0.05 **p ≤ 0.01 ***p ≤ 0.001 indicate Akt activator vs. vehicle and +*p* ≤ 0.05 indicates AR agonist after decomposition of the CSxTreatment interaction. **c** Effect of an acute administration of an anti-Akt drug or ERs agonist after FA on fear memory consolidation in osanetant treated proestrus females (vehicle: *n* = 6; anti-Akt: *n* = 7; ERs antagonist *n* = 5) (against vehicle: anti-Akt *p* = 0.000; ERs antagonist *p* = 0.003). *p ≤ 0.05 **p ≤ 0.01 ***p ≤ 0.001 indicate main treatment effect in repeated measures ANOVA and pairwise comparisons. Data are mean ± SEM. Main effect treatment, main effect CS, and CSxTreatment interaction were analyzed using repeated measures ANOVA. Two-tailed pairwise comparisons are indicated for (**b**) and (**c**).

As previously hypothesized, males that received systemic osanetant together with intra-CeA AR agonist (CI-4AS-1) or an Akt activator (SC 79) presented increased memory consolidation in comparison to those that only received osanetant, as shown by higher freezing rates in the expression test 24 h later (Fig. 6a, b, CS x Treatment interaction *F*_(1,8)_ = 4.107, *p* = 0.018; CS1-5: vehicle vs. Akt activator *p* = 0.003, vehicle vs. AR agonist *p* = 0.018; CS6-10: vehicle vs Akt activator *p* = 0.137, vehicle vs. AR agonist *p* = 0.010; CS11-15: vehicle vs Akt activator *p* = 0.031, vehicle vs AR agonist *p* = 0.021; Supplementary Fig. 8a). In contrast, proestrous females that received intra-CeA ERs antagonist (ICI 182,780) or an Akt inhibitor (Akti-1/2) together with systemic osanetant presented lower memory consolidation when compared to those proestrous females that only received osanetant, as expressed by lower freezing rates in the expression test 24 h after receiving the treatment (Fig. 6a, c, *F*_(1,15)_ = 21.299, *p* < 0.000, η2p = 0.740, vehicle vs. antiAkt *p* < 0.000, vehicle vs. ERs antagonist *p* = 0.003, antiAkt vs. ERs antagonist *p* = 0.021; Supplementary Fig. 8b).

In line with our hypothesis, local AR or Akt activation normalized the decreased freezing rates caused by the blockade of Nk3R by osanetant in male mice. In contrast, local blockade of ERs or Akt in female mice during proestrus reverted the effect of osanetant increasing fear memory consolidation in female mice.

## Discussion

**Fig. 7:**
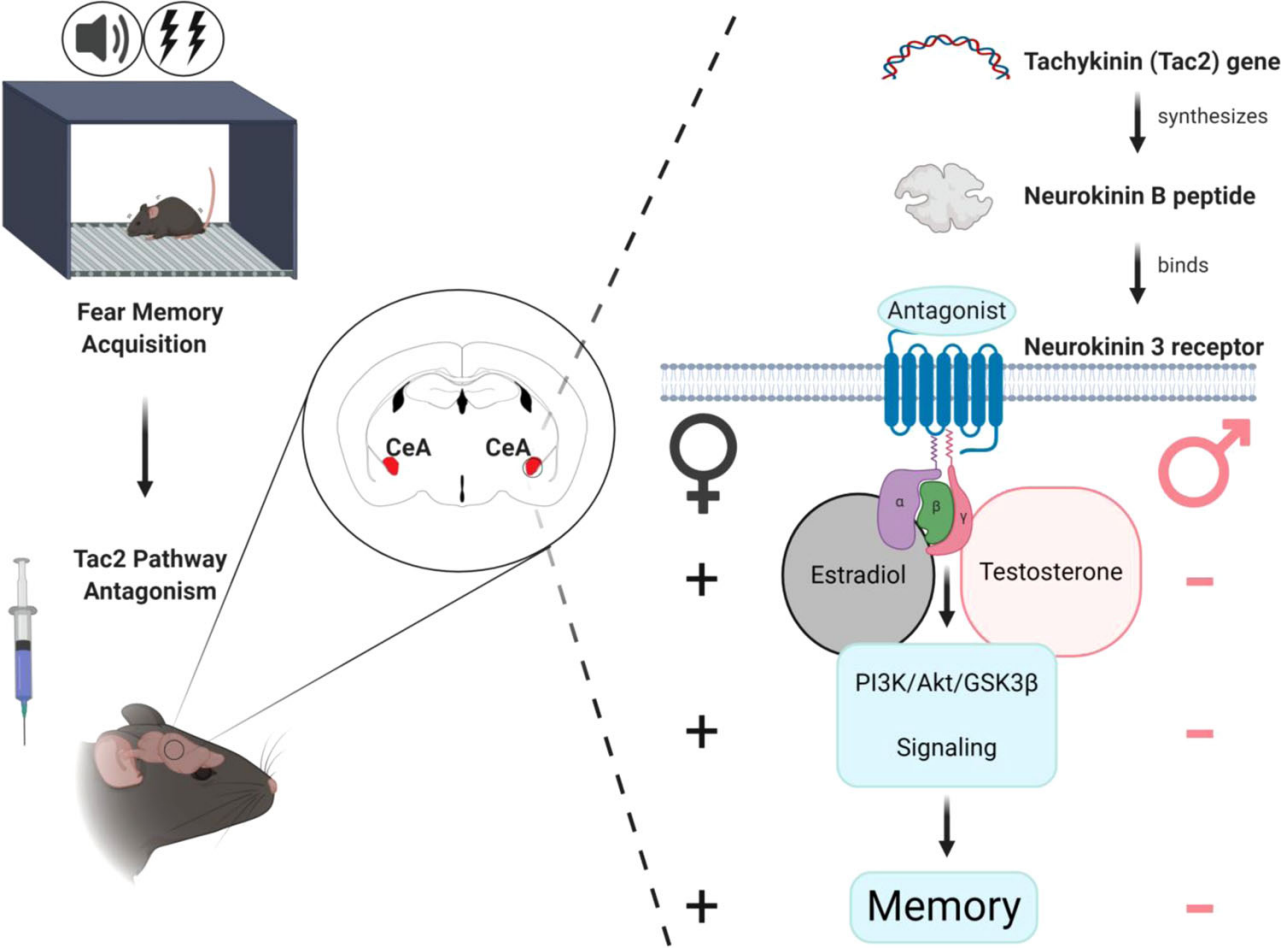
Graphical summary. A graphical overview of this study.

Taken together, our data show that the temporal inactivation of the CeA-Tac2 pathway results in impaired fear memory consolidation in males but increases fear memory in females. This memory effect is dependent on sex hormones and their specific receptors in the CeA; testosterone in males and estradiol in females. Additionally, Tac2 pathway inactivation is sufficient for amygdala Akt/GSK3β/β-Catenin signaling downregulation in males and upregulation in proestrous females, being these molecular changes necessary to reduce fear memory consolidation in adult males and increase it in adult females during proestrus. See Fig. 7 for a graphical summary of the results.

The study of sex differences in fear memory has vastly increased in the last years. Indeed, numerous previous studies have found sex differences in long-term memory formation and its mechanisms^4^. It has recently been reported greater contextual fear conditioning and generalization in females compared to males, with context pre-exposure increasing conditioning in adult males and decreasing generalization in females^28^. These results go in line with important differences in c-fos activation between males and females during memory retrieval and context generalization, with males showing increased c-fos activation in the dorsal hippocampus, while females in the basolateral amygdala^28^. Further, it has been described increased contextual fear conditioning in adult males in comparison to females as measured by freezing behavior, correlating with increased Long-Term Potentiation (LTP) in the dentate gyrus of the hippocampus^29^. Quite importantly, one of the possible reasons why adult male mice typically exhibit more freezing than females has been recently uncovered, and that is the nature of fear responses, with female mice exhibiting more active fear responses that directly compete with passive fear responses such as freezing in male mice^17^. Focusing on the molecular mechanisms of sex differences in fear memory, cyclin-dependent kinase^5^ (CDK5) mRNA and histone H3lysine 9/14 acetylation in the CA1 region of the hippocampus has been shown to be upregulated in adult male mice but not in females (with the estrous cycle unmonitored) using a contextual fear conditioning paradigm^30^. Other studies have focused on LTP an electrophysiological correlate of synaptic efficacy required for long-term memory consolidation^31^. Adult male and female rats show equivalent LTP, although the proven mechanisms in females involve cAMP-activated protein kinase (PKA) for the initiation of potentiation induced by estradiol whereas PKA is not necessary for the estradiol-induced potentiation in males^32^. Our current study shows a sex hormone-dependent modulation of memory consolidation, since testosterone results necessary for osanetant to reduce fear memory consolidation while the increase in estradiol levels in females is necessary for the enhancement of memory consolidation, as shown by the blockade of effects when we pharmacologically reverted the hormonal fluctuations caused by osanetant. Intraperitoneal injection of a dopamine receptor 1 agonist in adult female rats during low estradiol phases enhances fear memory recall whereas females trained during high estradiol estrous phases present impaired FE recall^33^. However, our data provide evidence that there are opposite-sex effects in behavioral and molecular mechanisms (Akt/GSK3β/β-Catenin signaling) of fear memory consolidation after a drug administration in the amygdala of adult mice.

### Dominance/submission, estrous cycle, hormones, and fear memory

Typically, the manipulations in rodent fear memory studies only involve one sex and generally do not account for traits or hormonal status which are relevant for brain functioning^9^. In females, it is only when osanetant is given during the proestrous stage that fear memory consolidation is enhanced. These results suggest a key role of circulating sex hormones in females mediating the effect of osanetant on fear memory consolidation. In line with previous studies that demonstrate an interaction between fear memory consolidation and the estrous cycle, it has been already reviewed that adult female rodents in proestrus present enhanced fear extinction retention^20,34^. Moreover, we have tested the effect of an Nk3R antagonist on fear memory consolidation depending on both dominance/submission in males and females and the estrous cycle in females. Regarding the social hierarchy, which is highly dependent on androgens in male and female mice^35–37^, results suggest that dominance status as assessed by CTT in males or females is not relevant for the modulation of memory consolidation induced by osanetant, thus suggesting a lack of effect of relatively basal testosterone levels in the modulation of osanetant effects on fear memory consolidation. However, an alternative explanation is that the stress associated with the evaluation of dominance/submission in rodents interferes with subsequent memory formation processes. Although animals do not present increased corticosterone concentration in serum before the FE test, we cannot discard an effect of stress because adrenocorticotropin releasing hormone (ACTH) or corticotropin-releasing factor (CRF) were not measured in our experiments. Moreover, in line with previous findings^38^, serum corticosterone in adult female mice is increased in relatively basal conditions in submissive females compared to the dominants^38^, but not in adult males, where slightly higher values of corticosterone in dominant versus submissive mice have been previously reported^39^. The use of different strains of mice or methodologies to assess hierarchies might explain this discrepancy between the reported data in males and the ones showed here. Noteworthy, this effect in females’ relatively basal corticosterone is abolished once the animals are habituated to a novel context such as the fear chamber. This exposition to a relatively mild stressor such as a novel environment might mitigate the observed differences in corticosterone between dominant and submissive mice. Further, and supported by existing literature^38^, social hierarchies remained quite stable along the different sessions of the CTT, and therefore we conclude there is not an effect of the estrous cycle in the determination of the dominant/submissive female. However, these differences do not appear relevant to modulate the enhancement of fear memory consolidation induced by osanetant.

Also, we found that our mice did not present a conditioned darting response, nor a difference between males and females in the dart count in response to the CS. These results contrast with previously reported conditioned darting behavior in adult female rats^17^, although some authors reported non-relevant levels of darting behavior during fear conditioning^40,41^. Furthermore, regarding healthy adult women, it has been previously demonstrated that a higher concentration of circulating estradiol enhances extinction recall, without an effect on FA or fear extinction itself^34^. However, we show that osanetant given after FA at different stages of the estrous cycle can result in different effects such as enhancement of memory or no effect. Remarkably, osanetant did not affect fear generalization to the context.

### Sex hormonal but not stress hormonal response to FA is altered by systemic osanetant

Our hormonal profile showed a testosterone modulation after FA with an initial decrease after fear acquisition and an increase hours later in those animals treated with vehicle. Of note, mixed results are commonly reported regarding testosterone regulation after exposure to mild acute stressors such as the footshocks, that mice received in our experiments, as it shows to be highly dependent on the nature of the stressor, its intensity, and duration. Further, discrepancies appear when different species or strains are tested. In male and female adult Sprague-Dawley rats, restraint stress showed no alterations in plasma testosterone and estradiol, respectively, after stress^42^. Further, it has been reported a lack of effect on sex hormones 24 h after restraint and footshock stress^43^. Some authors report increased serum concentration of testosterone after chronic noise stress (85 dB)^44^ whereas others show a decrease after 4 days of chronic stress by paradoxical sleep deprivation, footshocks, or cold in adult rats^45^. Moreover, it has also been reported a reduction in plasma testosterone in restrain-stressed adult male Sprague-Dawley rats after 3 h of restraint, but not after 1 or 2 h^46^. Importantly for the interpretation of our results, immobilization stress in male C57BL/6 mice has shown to decrease serum testosterone during stress up to 360 min after the onset of the stressor^47^, suggesting a rapid suppression effect of testosterone synthesis in response to stress. Following the findings by Dong et al.^47^, we hypothesize that the rapid reduction observed in testosterone levels after acute stress might be due to a glucocorticoid-mediated suppression through a non-genomic mechanism.

It is noteworthy in our data that osanetant prevented the peak of testosterone in males 360 min after FA. This is not surprising since it has been previously shown that Nk3R antagonism in adult humans reduces circulating sex hormones^18^. Previous studies show no changes on estradiol after a mild acute stressor such as an elevated platform even 6 h after the offset of the stressor^48^. In contrast, other studies using swim stress found increased estradiol concentrations 6 h after stress inoculation in adult females^49^. From these results, it can be concluded that the nature and intensity of the stressor may be a key factor in the regulation of circulating estradiol. In our experiment, FA did not change the estradiol levels in the vehicle group in females, concordantly with previous studies that show unaltered estradiol concentration after mild acute stress. Also, osanetant produced an increase in serum estradiol in proestrous females 6 h after FA (330 min after the administration) but not in metestrous females. Since a high variability is reported in circulating estradiol among species and strains^50–55^, we further confirmed our estradiol measurements by mass spectrometry (Supplementary Fig. 3e), similar to those values reported by others using the same ELISA kit^56^. Of note, progesterone was increased in males and decreased in proestrous females 1 h after FA, and remained unaltered in metestrous females, with no differences between vehicle and osanetant treated groups in any of the cases mentioned. Thus, we can discard the role of progesterone in the CeA-Tac2 pathway for fear memory consolidation. In line with the beforementioned results, our studies with cannulated animals demonstrate that a single intra-CeA dose of an AR agonist blocks the effect of osanetant to reduce fear memory consolidation in male mice. Furthermore, the infusion into the CeA of a dual ERs antagonist together with systemic osanetant inhibits the increase in memory consolidation in proestrous female mice. Altogether, these experiments show the necessary role of AR inactivation in males and ERs activation in females for osanetant to modulate memory consolidation in males and females.

Regarding stress hormones, it is known that circulating corticosterone is increased after mild stressors^57^. Further, it has been previously shown that Tac2 levels are increased after chronic stress exposure in adult male mice^58^. However, we rule out the role of the stress response in our FA experiments by showing no differences in corticosterone and related hormones after osanetant injection during the consolidation window of fear memories. Because ACTH and CRF levels have not been taken into account, we cannot discard their effects modulating the response to osanetant.

### GAD65 and CaMKIIα expression in CeA-Nk3R-positive neurons

Here, we demonstrate that there are no differences in the number of Nk3R-positive neurons in the CeA between males and females neither in proestrus nor metestrus. Thus, we discard the possibility for a distinct number of CeA-Nk3R-positive neurons mediating the effects of osanetant in males and females in fear memory consolidation. Notwithstanding, there are important physiological differences in these neurons in terms of colocalization with enzymes involved in synapse function/plasticity such as GAD65 and CaMKIIα.

GAD65 is one of the main enzymes that synthesize γ-Aminobutyric Acid (GABA) in the brain, together with GAD67. There have been previously reported sex differences in GAD65 synthesis, with increased synthesis in adult female rats dorsomedial hypothalamus and medial amygdala, while GAD67 appeared to be higher in males^59^. GABAergic transmission is abundant in the CeA, and key in GABA-dependent synaptic plasticity^60–62^. Previous studies have highlighted that estradiol increases GAD65 mRNA while decreases GAD67 mRNA as measured by in situ hybridization in adult rat brain areas like the magnocellular preoptic area or the dorsomedial hypothalamus^63^, but not in the amygdala. Further, it has been proven that estradiol is necessary for GAD enzymes to produce GABA in adult females^64^. Interestingly, we are not aware of reports studying sex differences in GABAergic transmission in the CeA nor its role in fear memory. One of the tested hypotheses was that Nk3R could be expressed differentially in excitatory and inhibitory neurons, and thus, it could influence in totally opposite ways the consolidation of fear memory in males and females. Herein, we found enhanced colocalization of central amygdala Nk3R in GAD65-positive neurons in females exclusively during the proestrous stage of the estrous cycle in comparison to metestrous females and males, that present similar levels of colocalization. These data suggest the possibility of Nk3R to regulate GABAergic-dependent synaptic plasticity in proestrous females.

CaMKIIα is a postsynaptic effector critical for memory because of its involvement in long-term potentiation through phosphorylation of α-amino-3-hydroxy-5-methyl-4-isoxazolepropionic acid (AMPA) receptors^65,66^. In the CeA, CaMKIIα is expressed in cell bodies and neuropil of glutamatergic neurons^67^. To the best of our knowledge, no studies have addressed differences in CaMKIIα expression between males and females in the amygdala. Here, proestrous and metestrous females showed increased colocalization of CaMKIIα with Nk3R in the CeA in comparison to males. This result may indicate a tendency to increased CaMKIIα-dependent synaptic plasticity related to fear memories in females than in males.

### The CeA Akt/GSK3β/β-Catenin pathway is oppositely regulated between sexes in memory consolidation

The Akt/GSK3β/β-Catenin pathway is widely known for its role in amygdalar- and hippocampal-dependent learning and memory^68,69^ and cognitive flexibility^70^. Of note, hippocampal memory has shown to require estradiol for PI3K activation^71^. PI3K/Akt is upstream of important memory targets like mTOR, CREB, and GSK3β^72^. The PI3K/Akt/GSK3β pathway has been evidenced for its role in cued-fear memory consolidation through stabilization of β-Catenin^73^. Further, there is increasing evidence of inhibitory interactions between estradiol and GSK3β towards the β-Catenin stabilization through ERα promoting memory in the hippocampus^74^.

In our study, we show an opposite-sex regulation of the Akt/GSK3β/β-Catenin pathway in males and proestrous females treated with osanetant when high serum estradiol is present during the consolidation window of fear memory. Our results show an increase of GPCR-related genes in females during proestrus after FA and treatment with osanetant, and a decrease of GPCR-related genes in males after the same conditions, in line with the behavioral outcome. Also, bioinformatics analyses of GPCR altered genes pointed at the Akt pathway as the central/key modulator of the effects of the drug. To further understand the involvement of Akt in the behavioral effects mentioned, we used biochemical approaches to analyze the different downstream targets of Akt. Although we found no significant alterations of CREB and mTOR, two well-known factors involved in synaptic plasticity and memory, we cannot discard them as contributors to the osanetant-induced effect in memory consolidation because of different methodologies or time points may show different results. Notwithstanding, we show in CeA a decrease in total GSK3β levels in males while, in females, total Akt and phosphorylated GSK3β (Ser9; inactive GSK3β) are increased in response to the drug after FA, providing a potential target of osanetant in the modulation of fear memory consolidation in male and female mice. In line with GSK3β inactivation, and because β-Catenin is a direct downstream target of GSK3β, phosphorylated β-Catenin at residues Ser33/37/Thr41 targeted by GSK3β is reduced towards a clear tendency to elevated β-Catenin in female mice. This result agrees with previous findings indicating a role of β-Catenin in cued-fear memory consolidation^73^, suggesting that activation of Akt/GSK3β/β-Catenin mediates long-term stabilization of the recently acquired memory in adult male and female mice. Besides, we show in males that the pharmacological activation of Akt in the CeA, together with systemic osanetant, blocks the effects of the Nk3R antagonist to decrease fear memory consolidation. In contrast, in females during proestrus, the local administration in the CeA of an anti-Akt blocks the effects of osanetant of increasing memory consolidation. Altogether, these results evidence the opposing role between sexes on Akt/GSK3β/β-Catenin signaling in the regulation of amygdala-dependent memory consolidation.

As previously reported^10^, there is a reduction of fear memory consolidation by Nk3R inactivation in males, in line with what is published and expected. Notwithstanding, blocking Nk3R in females increases Akt/GSK3β/β-Catenin signaling which suggests that this pathway mediates the increase in consolidation observed in behavior. In line with the immunohistochemistry assays described above, in which Nk3R/CaMKIIα colocalization is upregulated in females in comparison to males, it is possible that the increase observed in memory consolidation could be partially dependent on CaMKIIα, which is part of a different molecular pathway related to PKA and CREB.

These data thereby provide evidence that there exist opposite-sex effects after a drug administration in behavioral and molecular mechanisms in memory formation through the involvement of the Akt/GSK3-β/β-Catenin signaling pathway in Nk3R-positive neurons of the CeA in interaction with sex hormones. Thus, our discovery proposes a diverse perspective on why and how memory processes differ between females and males.

## Methods

### Ethics and biosecurity protocols

Ethics protocols approved for the experiments in mice ref. CEEAH 3603 and biosecurity protocols 345-16 and 407-17. All procedures were approved by the Committee of Ethics of the Universitat Autònoma de Barcelona and the Generalitat de Catalunya. They were also carried out in accordance with the European Communities Council Directive (2010-63-UE) and Spanish legislation (RD 53/2013).

### Mice

All experiments used male and naturally cycling female adult mice (8 weeks old has been used for all the experiments except, P28 for prepubertal mice and 2–6 months of age for inbred Tac2-Cre mice) housed in groups of 4 (except when mentioned) in a room with a 12:12 h light/dark cycle (lights on from 8 am to 8 pm). All animals were housed with a controlled temperature of 22 ± 1 °C and humidity (~40%). Behavioral procedures and pharmacological manipulations began early in the light phase of the cycle. B6.129-Tac2 <tm1.1(cre)Qima >/J (Tac2-cre) (stock# 018938 Jackson Labs) were bred within our animal facility. Wild-type C57BL/6J were purchased from Charles River (Barcelona, Spain). Male and female mice were housed separately in the same room.

### Cued-Fear Conditioning

For Fear Acquisition (FA) and Fear Expression (FE) test, a computerized Startle system was employed (Panlab-Harvard, Barcelona, Spain)^75^. Delivery of tones and shocks was simultaneously controlled by Freezing v1.3.04 software (Panlab-Harvard, Barcelona, Spain). The fear chamber consisted of a black methacrylate box with a transparent front door (25 × 25 × 25 cm^3^) inside a sound-attenuating chamber (67 × 53 × 55 cm^3^). The same boxes were used for FA and FE.

Animals were habituated to the chambers for 5 min/day for two consecutive days before FA. For cue-dependent fear conditioning, all animals remained 5 min in the fear chamber before the onset of the first tone. During FA, all groups received 5 trials consisting of a tone as the Conditioned Stimulus (CS) (30 s, 6 kHz, 75 dB) that coterminated with a footshock which served as the Unconditioned Stimulus (US) (1 s, 0.3 mA). The intertrial interval (ITI) was 3 min, and 3 additional min followed the last trial. All animals received the treatments 30 min after FA to manipulate the consolidation of memory and therefore avoid any effect during acquisition. The FE test was performed 24 h after FA. Mice remained 5 min in the chamber before trials and afterward were exposed to 15 trials of the 30 s CS tone alone (cued-fear) with a 0.5 min of ITI interval. An additional 0.5 min interval followed the last trial of FE. Freezing behavior was used as an index of fear. Freezing behavior understood as a lack of any movement of the animal but for those related to breathing, was recorded using the StartFear system, which allows recording and analysis of the signal generated by the animal movement through a high sensitivity weight transducer system. To that end, a movement threshold was established for low mobility (13/100) and a freezing episode was considered when the animal spent at least 500 ms under this threshold. This system was validated by 5 experimented researchers through stopwatch analyses of video recordings. Darting behavior, as known as rapid peaks of locomotor behavior much above the normal locomotor activity of the mouse was counted by an experimented observer^17^. These recordings were performed throughout the session and separated in bouts of 5 min of habituation and then, independently, during each CS and ITI.

To assure that freezing behavior was exclusive of the previous tone-shock conditioning, different contexts were utilized for FA and FE. FA context consisted of a yellow light source (~10 lux), a grid floor of 25 bars (3 mm Ø and 10 mm between bars) that dispensed the footshocks, background noise of 60 dB produced by a ventilation fan and a solution of ethanol (EtOH) 70% (v/v) odor was used for cleaning between sessions. FE context consisted of a red light source (~10 lux), a gray floor covering the bars, no background noise, and CR36—bronopol 0.26 % (v/v), benzalkonium chloride 0.08% (v/v), and isopropyl alcohol 41% (v/v)—(José Collado, Barcelona, Spain) for cleaning, with changes in the length and turns of the transportation route from the vivarium to the testing room between FA and FE.

### Confrontation tube test (CTT)

Female and male mice were pair-housed for 5 weeks before testing to establish stable social hierarchies between them. The confrontation tube consisted of a methacrylate tube (30 cm length, 3.6 cm inner Ø) with two lids at 13 cm from each end. All animals were habituated to the tube for 2 consecutive days before the test. The habituation consisted of 3 crossings from one end to the other of the tube and the tube was always cleaned with EtOH 70% (v/v) between animals. Mice were tested for 6 consecutive days, with 8 trials per day with ~20 min between trials. For each trial, each mouse from each couple entered the tube simultaneously by the extremes. When both animals reached the lids, these were lifted for animals to face each other and that’s when the test started. The first animal that put its four limbs outside the tube was considered the loser while the other one won the trial. When both animals remained 2 min inside the tube without confronting each other, the trial was considered null. The animal that presented more won trials throughout each session was considered the winner of the session. At the end of the six sessions, the animal that presented more won sessions was considered dominant over its cage mate. The day after the 6th session of the tube test, animals underwent the FC protocol as abovementioned. All mice received osanetant 30 min after FA and were tested for memory recall 24 h later to assess for differences in the effect of the drug between dominant and submissive mice.

We extracted 50 μl of blood from each animal by tail-nick two days before the first habituation to the tube (under relatively basal conditions), 2 h before the first habituation session, 2 h before the first test and 2 h before the first habituation to the fear conditioning chamber, thus, with every two days since the first extraction. Blood was centrifuged (8000 *g*, 15 min, 4 °C) and serum was stored at −80 °C. In female mice, we also monitored the estrous cycle daily from the first habituation to the tail-nick extraction until the FE test.

### Vaginal smear cytologies

Determination of the estrous stage in females was performed by assessing vaginal smear cytologies^76,77^. To assess the phase of the estrous cycle that female mice presented during FA, all female mice were monitored for 3–4 consecutive cycles (approximately 10-14 days) before learning to test for regularity of the cycle. We performed a vaginal lavage with a 20 μl pipette that was loaded with 10 μl of standard NaCl 0.9% (w/v) solution, and later the tip was softly placed on the vaginal aperture. In case of urination when grabbing the animal, urine was cleaned using a regular tissue. The 10 μl of saline were unloaded and collected for 5 consecutive times to collect enough amount cells for the assessment, and later placed on an adhesion slide (Superfrost Plus, Thermo Fisher, Barcelona, Spain). All vaginal smear samples were collected between 9:30 and 11:30 am. Slides were dried using a hot plate (HI1220, Leica, Madrid, Spain) at 37 °C for 30 min and later stained in Cresyl Violet Acetate (C5042, Sigma-Aldrich, Spain) 0.1% (v/v), washed twice for 1 min in distilled water and read in brightfield microscopy with a 10× or 20× objective in an Eclipse 80i microscope (Zeiss, Spain). Three different cell types may appear in the preparation: cornified epithelial cells, round nucleated epithelial cells, or leukocytes. The different stages of the estrous cycle were assessed depending on the proportion of the abovementioned cells. Proestrus is characterized by a high proportion (>80%) of nucleated epithelial cells, that might present very small amounts of cornified epithelial cells or leukocytes. Estrus is typically presented with cornified epithelial cells with a lower grade of staining than leukocytes and nucleated epithelial cells. Metestrus presents a mixture of cornified epithelial cells and a considerable proportion of leukocytes. Diestrus is characterized by >90% of leukocytes that might present a very small proportion of round nucleated epithelial cells. After assessment of regular cycling, females were distributed in groups according to the stage of the estrous cycle they presented before FA, which is also the day they receive the different treatments after FA.

### Drugs

Intraperitoneal osanetant (Sigma-Aldrich, Spain) dose was 5 mg/Kg 10, 1 mg/Kg or 10 mg/Kg, and the intracerebral dose was 30 nmol per side as it has shown to modulate fear memory consolidation in male mice when administered intra-Central amygdala (CeA)^10^ using 0.1% Tween 20 in saline as the vehicle. The dose of intra-CeA Androgen Receptor (AR) agonist CI-4AS-1 (Tocris, UK) was 100 nM based on previous research showing decreased depolarization-induced suppression of excitation in POMC neurons^78^, dissolved in 1% DMSO (v/v). We used the AR agonist instead of testosterone to avoid its possible conversion to 17-β-estradiol by aromatase. The Akt activator SC^79^ (Tocris, UK) was used at a dose of 0.1 µM in 1% DMSO (v/v) because it has shown to have a protective effect on dopaminergic neurons against oxidative stress^79^. Anti-Akt1/2 (Tocris, UK) was used at 5 µM in 1% DMSO (v/v) due to its ability to block Akt phosphorylation^80^. Estrogen Receptors (ERs) antagonist ICI 182,780 (Tocris, UK) was used at a 10 µM in 1% DMSO (v/v) as it has shown previously to be effective in decreasing EtOH excitation of DA neurons in the VTA^81^. CNO in 0.5% DMSO (v/v) systemically was dosed at 1 mg/Kg^10^.

### Surgery

All surgeries were performed using isoflurane 5% (v/v) for induction, and 2–3% (v/v) for maintenance, in oxygen, at a constant rate of 1.5 l/min. After skin shave and skin disinfection with EtOH 95% (v/v) and iodine povidone 10% (v/v), ovariectomies were performed making a bilateral incision on the back of the animal, 1 cm lateral to the midline and right over the back limbs line. Adipose tissue was extracted, and the ovary was localized and isolated making a knot with sterile absorbable suture thread (Centauro, Spain) around the oviduct. The ovary was extirpated and the adipose tissue, containing the rest of the oviduct, was returned to the abdominal cavity. The muscle was sewed with sterile absorbable thread and skin was sewed using sterile silk suture (Centauro, Spain). Mice remained resting for 6 weeks until trunk blood was collected to avoid any effect of previous estradiol.

For stereotaxic surgeries, after induction of anesthesia, mice were placed in the stereotaxic frame (Kopf Model 962, Harvard-Panlab, Barcelona, Spain). After alignment of the Antero-Posterior (AP) and Latero-Medial (LM) axis with the frame, injections of AAV8-hSyn-DIO-hM4D(Gi)-mCherry were performed at a rate of 1 μl per 15 min to the CeA using the following coordinates: AP −1.3, LM ± 2.5, Dorso-Ventral (DV) –4.4 mm from Bregma^10^. For cannulation, the same AP and ML coordinates were utilized, although cannulae were implanted at DV −3.4 mm due to the need to remain the CeA intact and because of the extra mm projection of the internal cannula. Cannulae were secured to the skull with anchor screws for mice (Plastics One, Germany) and dental cement (Fortex Fájula, Cibertec, Spain) For further details on the virus and microinfusions, please see Adeno associate virus (AAV) infection and microinfusions section below.

### Adeno-associate virus (AAV) infection and microinfusions

We used AAV obtained from the pAAV-hSyn-DIO-hM4D(Gi)-mCherry (hM4di-mCherry DREADDs). The plasmid was obtained from Addgene and the AAV from the viral vector production unit at Universitat Autònoma de Barcelona. Serotype 8 of the AAV was bilaterally injected (0.5 μl/side) using a microinjection pump into the CeA (coordinates and infusion rate abovementioned). Cannulae were placed using coordinates mentioned in the surgery section. For microinfusions of drugs in awake animals, internal cannulae projected 1 mm from the tip of the internal guide, reaching DV coordinate −4.4 mm from Bregma for injections, corresponding to the CeA. Bilateral injections were performed simultaneously and manually using two knurled hub, type 3 tip, 1 μl Hamilton syringe (external Ø 0.52 mm and internal Ø 0.26 mm) (Cibertec-Harvard, Madrid, Spain) coupled on one end to a polyethylene (PE) 50 tube (~20 cm) (Plastics One, Germany) with the internal cannula coupled to the other end of the tube. The PE-50 tube was filled with distilled water, leaving a 7.5 mm gap of air between the internal cannula and the water to avoid dilution of the drugs with water. Microinfusions were performed at a rate of 0.5 μl over 2 min and internal cannulae remained one extra min in place to prevent the backflow of the drug.

### Steroids determination

For steroids determination in Fig. 3, trunk blood was collected after sacrificing the animals. We used animals under relatively basal conditions that did not undergo any procedure whose blood was collected within 3 min of touching the homecage, animals with FA and an injection of osanetant 30 min after that were euthanized 30 min after receiving the injection, and animals with FA and osanetant 30 min after that were euthanized 330 min after the injection for the three cohorts of study: males, proestrous females, and metestrous females. Corticosterone determination was performed 30 min after receiving osanetant or vehicle since these hormones have shown to be altered after fear conditioning procedures^57^. While we chose 330 min after treatments for estradiol assessment since previous studies have shown a long-term (4–6 h) regulation of this hormone after stress^48,49^. For Fig. 2, males and females’ blood was collected by tail-nick as described in the CTT section. Blood was centrifuged (8000 *g*, 15 min, 4 °C). Serum was collected using a 200 µl pipette, transferred into a 2.5 ml Eppendorf tube, and stored at −80 °C for later analysis. Brains from animals euthanized 330 min after treatment (6 h after FA) were snap-frozen using Isopentane cooled with dry ice, and later stored at −80 °C until sectioning. Fresh frozen brains were then coronally sectioned until reaching a coronal plane 0.58 mm behind Bregma. 1 mm Ø micropunches from both Amygdalae were extracted reaching coronal plane 1.94 mm behind Bregma, transferred into a 2.5 ml Eppendorf tube, and stored until processing. 400 μl of Formic Acid (Sigma-Aldrich, Spain) 0.1% (v/v) were added to each tube and sonicated for five consecutive cycles. The sonication probe was then rinsed with 800 μl of Acetonitrile (Sigma-Aldrich, Spain) that were added to the tubes, and these were vortexed for 2 s. Micropunches extracts were stored at −80 °C until analysis. Both serum and amygdalar testosterone, progesterone, dehydrocorticosterone, and deoxycorticosterone were evaluated based on previously reported papers^82,83^. Briefly, 20 µl of plasma was mixed with 20 µl of a labeled internal standard solution. After protein precipitation with 100 µl of acetonitrile, samples were centrifuged (3000*g*, 5 min) and the supernatant transferred to a clean tube. In the case of micropunches 20 µl of the labeled internal standard solution was added to the 1 ml of extract from micropunches. The mixture was vortexed and transferred to a clean tube. Both plasma and micropunches underwent a liquid-liquid extraction by the addition of 1 ml of NaCl (saturated solution) and 4 ml of ethyl acetate. Extracts were centrifuged (3000 *g*, 5 min) and the organic layer was transferred into a clean tube and dried under a nitrogen stream. Dried extracts were reconstituted with 100 µl of methanol and 10 µl were injected into the LC-MS/MS system consisting of an Acquity UPLC system coupled to a triple quadrupole (TQS Micro) mass spectrometer. Steroids detection was performed by selected reaction monitoring (SRM) including two transitions for each analyte. The most specific one was selected for the quantification. Quantification was performed by an external calibration approach using the TargetLynx module of the MassLynx v4.1 software (Water Associates, USA). Estradiol was measured with the ELISA kit ES180S-100 (Calbiotech, California, USA). Ovariectomized (OVX) mice were used for the ELISA analyses of estradiol with the ELISA kit. For standard curve preparation, serum from OVX mice was used adding known concentrations of estradiol (Sigma-Aldrich, Spain)—0, 3, 10, 30, 100, and 300 ρg/ml—utilizing denaturalized EtOH (Casa Álvarez, Spain) as a vector for estradiol. Kit instructions were followed as stated, samples were loaded in duplicates and absorbance was read at 450 nm with the microplate reader Varioskan Lux (Thermo Fisher, Spain) controlled with Skanit for microplates v6.0 software (Thermo Fisher, Spain). The average of duplicates was used as estradiol determinations for each sample.

Estradiol results were confirmed by the LC-MS/MS method based on chemical derivatization and detection in the SRM mode. Briefly, plasma samples (c.a. 100 µl) were mixed with 20 µl of internal standard (estradiol-d3 at 2 ng/ml) and 1 ml of water and were subjected to two consecutive liquid-liquid extractions with tert-butyl methyl ether. The organic layers were mixed and evaporated under nitrogen stream (<40 °C, < 15 psi). Derivatization with 1,2-Dimethylimidazole-5-sulfonyl chloride was performed at 60 °C for 15 min. Derivatized extracts were evaporated under nitrogen stream (<40 °C, < 15 psi) and reconstituted in 1 ml of water. Two additional liquid-liquid extractions with 4 ml of hexane were performed. The organic layers were mixed and evaporated under nitrogen stream (<40 °C, < 15 psi) and the extracts were reconstituted in 100 µl water:methanol (50:50). Ten microliters were injected into the LC-MS/MS system consisting of an Acquity UPLC system coupled to a triple quadrupole (TQS Micro) mass spectrometer. Chromatographic separation was performed in an Acquity BEH C18 column (100 mm × 2.1 mm i.d., 1.7 μm) (Waters Associates) at 55 °C and at a flow rate of 300 μl/min. Mobile phases consisted in water with ammonium formate (1 mM) and formic acid (0.01 % v/v) and methanol with ammonium formate (1 mM) and formic acid (0.01 % v/v). The selected gradient program linearly changed the organic solvent percentage as follows: 0 min, 65%; 1 min, 65%; 3.7 min, 68%; 3.8 min, 99%; 4.8 min, 99%; 4.9 min, 65%; 6.0 min, 65%. Quantification of estradiol was performed in the SRM mode by monitoring the transitions of 431 > 96, 431 > 161 and 431 > 367 for estradiol and 434 > 96, 434 > 161 and 434 > 370 for estradiol-d3.

### Immunohistochemistry

For immunohistochemistry assays, mice were transcardially perfused with 50 ml of 4% (v/v) paraformaldehyde (PFA) (Casa Álvarez, Spain) for 5–6 min, then decapitated, and brains were extracted and stored in 4% (v/v) PFA for 24 h. After this time, brains were rinsed (3 times, 10 min each) with 1× Sorenson’s PB consisting of 10.9 g/l Sodium phosphate dibasic (Sigma-Aldrich, Spain), 3.2 g/L Sodium phosphate monobasic (Sigma-Aldrich, Spain) and transferred into 30% Sucrose (Sigma-Aldrich, Spain) in 1× Sorenson’s PB in conic Falcon tubes (Thermo Fisher, Spain) until the brain reached the bottom of the tube (~48–72 h). Right after, brains were snap-frozen in a metal cube containing Isopentane (Sigma-Aldrich, Spain) cooled with dry ice and stored at −80 °C until sectioning.

For Neurokinin 3 Receptor (Nk3R), Glutamic Acid Decarboxylase 65 (GAD65), Calmodulin Kinase II α (CaMKIIα), and Vesicular Glutamate Transporter 2 (vGLUT2) fluorescent immunostaining brain sections (30 μm/section) were rinsed three consecutive times with 1× KPBS. All incubations were performed on top of a shaking platform. Right after washing the slices, these were incubated for 60 min in blocking buffer (5% Donkey Serum and 0.4% Triton-X in 1× KPBS) at 4 °C. After incubation in blocking buffer, sections were incubated overnight with the following primary antibodies: rabbit anti-Nk3R (Donated by Philip Cioffi, INSERM, 1:1500), chicken anti-GAD65 (139958, Abcam, 1:500), goat anti-CaMKIIα (87597, Abcam, 1:500) and mouse monoclonal anti-vGlut2 (ab79157, Abcam, 1:300). Primary antibodies were diluted in 0.4% Triton-X in 1× KPBS. After incubation in primary antibodies solution, brain slices were rinsed 3 times with KPBS 1× and then incubated in a secondary antibodies solution for 2 h at room temperature. The secondary antibodies solution was prepared in Triton X (×100, Sigma-Aldrich, Spain) 0.4% (v/v) in 1× KPBS, and included the following secondary antibodies conjugated to fluorophores: donkey anti-rabbit AlexaFluor488 (115-546-072, Jackson Immunoresearch, 1:1000), donkey anti-chicken Cyanine3 (703-166-155, Jackson Immunoresearch, 1:1000), donkey anti-goat AlexaFluor594 (705-586-147, Jackson Immunoresearch, 1:1000) and donkey anti-mouse AlexaFluor647 (715-606-150, Jackson Immunoresearch, 1:1000). 4′,6-diamidino-2-phenylindole (DAPI) (10236276001, Sigma-Aldrich, 1:10000) was used to stain cell nuclei.

Z-Stacks of the central amygdala (0.50 μm/interval) were acquired using a Leica SP5 confocal microscope (Leica, Spain) with a PL APO 40x/1.25-0.75 immersion objective controlled by LAS X v2.7.3.9729 software (Leica, Spain). Nk3R-positive neurons were determined using the cell count plugin from Fiji for Windows v1.52.n. Colocalization analyses were performed using Fiji for Windows v1.52.n. Z-Projections of Nk3R signal was used to create a mask. This mask was then applied to Z-Projections (0.50 μm/interval) of GAD65, CaMKIIα, and vGLUT2 stacks using LungJ plugin. Mander’s colocalization coefficient between Nk3R and GAD65, CaMKIIα, and vGLUT2 was calculated using Just Another Colocalization Plugin (JACoP). Average colocalization index between right and left CeA was used as a measure of colocalization for each animal.

For Estrogen Receptor β (ERβ) and Estrogen Receptor α (ERα) fluorescent immunostaining, the staining protocol was similar to the one mentioned above. Primary antibody solution contained rabbit anti-Nk3R (Donated by Philip Cioffi, 1:1500) and mouse monoclonal anti- ERα (sc-8002, Santa Cruz, 1:50) or mouse monoclonal anti- ERβ (288, Abcam, 1:500). Secondary antibodies solution included donkey anti-rabbit AlexaFluor488 (115-546-072, Jackson Immunoresearch, 1:1000) and donkey anti-mouse Rhodamine Red-X (715-295-150, Jackson Immunoresearch, 1:1000). DAPI (Sigma-Aldrich #10236276001) was used to stain cell nuclei. Z-Stacks of the central amygdala (0.50 μm/interval) were acquired using a Zeiss LSM 700 confocal microscope (Zeiss, Spain) with a PL APO 40x/1.25-0.75 immersion objective controlled by ZEN v2010 software (Zeiss, Spain). Cell counter plugin for Fiji v1.52.n was employed to measure the number of Nk3R-positive cells in the CeM. Mander’s colocalization coefficients for ERα and ERβ were calculated as abovementioned for Nk3R, GAD65, CaMKIIα and vGLUT2 immunohistochemistry using JACoP plugin in Fiji v1.52.n for Windows.

### Cannula placement verification

For cannula placement verification, amygdala brain sections were stained with a standard Nissl staining protocol. Brains were sectioned (30 μm/section) using a Leica Cryostat (−20 °C for the chamber, −18 °C for the sample), direct to mount and stored at −20 °C until staining. Slides were dried on a hot plate at 37 °C overnight before staining. First, slides were dehydrated in consecutive decreasing concentrations of denaturalized EtOH (Casa Álvarez, Spain): 3 min in EtOH 100% (v/v), 3 min in EtOH 95% (v/v) and 3 min in 70% (v/v). Right after that, slides were rinsed in distilled water three times for 2 min each to extract any trace of sucrose. Samples were then stained for 8 min in a Cresyl Violet Acetate Solution consisting of 1 mg/ml Cresyl Violet Acetate in Walpole Solution, prepared with Glacial Acetic Acid (Sigma-Aldrich, Spain) 60% (v/v) and Sodium Acetate 0.2 M (Sigma-Aldrich, Spain) 40% (v/v). After staining, slides are rinsed twice in distilled water for 2 min each and then dehydrated using increasing concentrations of EtOH: 3 dips in EtOH 50% (v/v), 3 dips in EtOH 70% (v/v), 3 dips in EtOH 96% (v/v) and 3 min in EtOH 100% (v/v). After this last dehydration, slides are incubated in Xylene (Sigma-Aldrich, Spain) 3 times for 3 min each and then covered using DPX mountant for histology (Sigma-Aldrich, Spain). Slides remain untouched for 24 h in the dark until stored in a regular box for slides at room temperature.

Cannulae placement verification was performed using brightfield photographs obtained using a 10x objective in an Eclipse 80i microscope (Leica, Spain). Animals that did not present a bilateral cannula lesion in the central amygdala were discarded from experiments.

### mRNA qPCR array

30 min after receiving osanetant (1 h after FA), animals were decapitated, and brains were immediately fresh frozen in isopentane cooled with dry ice and stored at −80 °C. Amygdala tissue from both hemispheres was extracted by 1 mm micropunch as previously described and each structure from each mouse was individually stored. Total RNA was isolated and purified from the tissue with the RNeasy Mini Kit (74106, Qiagen) following the manufacturer’s instructions. Total RNA was isolated with Maxwell RSC simplyRNA Tissue Kit (AS1390, Promega). Quantus Fluorometer (E6150 Promega) was used to ensure the quality of RNA before the qPCR array (PAMM-071ZC-24, Qiagen). Gusb was the housekeeping gene used for the normalization of qPCR results. qPCR array was performed following the instructions of the kit as stated, using the thermocycler 7500FAST, controlled by 7500FAST v2.0.6 software both from Applied Biosystems (Thermo Fisher, Spain).

### Ingenuity pathway analysis—bioinformatics

The gene list obtained on the mRNA qPCR array was analyzed with Bioinformatics. We used Ingenuity Pathway Analysis (IPA) software version #44691306. The male and female lists of genes selected according to their significant p-value were uploaded separately. All genes were introduced with their respective expression fold change value. The female’s gene list contained the genes *Akt1, Gcgr, Fgf2, Lhcgr, Mmp9, Ptgdr, Rho, S1pr3, Vcam1*. The males’ gene list contained the genes *Agt, Agtrap, Bcl2, Calcrl, Ccnd1, Ccne1, Cdkn1a, Cdkn1b, Cflar, Elk4, Galr2, Gnas, Bcl2l1*. We performed a Core Analysis/Expression Analysis using the fold change as a measurement to base the analysis on. These analyses were performed separately for males and females by using most of the predetermined criteria by IPA. The general settings of this analysis included both direct and indirect relationships based on the reference set “Ingenuity Knowledge Base (Genes Only)”. For the network analysis, we selected 35 molecules per network and 25 networks per analysis. We also choose all Node Type and all Data Sources. For Confidence, we selected only “Experimentally observed”. For Species we selected all. The results we obtained with this IPA analysis were focused on two categories: Canonical Pathways (Fig. 5b) and Upstream analysis (Supplementary Fig. 5b). For the Canonical Pathways, we customized the charts by using a criterion of only selecting the categories that presented more than 4.5-fold changes in both males and females. The graphic in Fig. 5b was created opening the PI3K/Akt in males and the G-coupled protein receptor signaling in females. Both graphics were combined using the Path Designer tool.

### Biochemical studies

After 10 min of receiving osanetant (40 min after FA), male and proestrous female mice were decapitated and brains were snap-frozen in isopentane right before storage at −80 °C. Also, an additional group of proestrous females and another of males that did not undergo fear conditioning nor osanetant injection were used as relatively basal animals. Both amygdalae were microdissected as abovementioned with a 1 mm micropunch, homogenized by sonication in 80 µl of cold-lysis buffer (50 mM Tris HCl, pH 7.4, 150 mM NaCl, 0.1% SDS, 1% NP-40, 0.5% Na-deoxycholate, 2.5 mM EDTA, 1 mM Na3VO4, 25 mM NaF) containing protease and phosphatase inhibitors (Roche España, Spain). Protein concentration was quantified with the BCA protein assay kit (Thermo Fisher Scientific, Barcelona, Spain), resolved on SDS-polyacrylamide gel electrophoresis, and detected by Western blotting with the following antibodies: rabbit anti-phosphorylated Akt (Thr308, 1:1000; Cell Signaling; Ser743, 1:1000; Cell Signaling) and goat anti-total Akt (1:1000; Santa Cruz Biotechnology), rabbit anti-CREB (1:700; Cell Signaling) and anti-phosphorylated CREB (Ser133; 1:1500; Cell Signaling), rabbit mTOR (1:1000; Cell Signaling) and anti-phosphorylated mTOR (Ser2448; 1:1000; Cell Signaling), mouse GSK3β (1:2500; BD Biosciences) and rabbit anti-phosphorylated GSK3β (Ser9; 1:1000; Cell Signaling), rabbit anti-β-Catenin (1:6000; Sigma-Aldrich) and anti-phosphorylated β-Catenin (Ser33/37/Thr41; 1:1000; Cell Signaling) or mouse anti-GAPDH (1:100000; Life Technology). Then, protein bands were detected with secondary antibodies coupled to peroxidase enzyme (Bio-Rad, Spain), and enhanced chemiluminescent reagent were captured in ChemiDoc MP System (Bio-Rad, Spain) and quantified in a linear range using the ImageLab v5.2.1 software (Bio-Rad, Spain) as reported^84^.

### Data analyses

Statistics analyses were performed using IBM SPSS Statistics 23.0. Detection of outliers was using Grubb’s test and removed when it was appropriate. For experiments involving independent samples, when these presented normal distributions and equality of variances, one-way ANOVA (GLM) was employed; otherwise, non-parametric analyses were utilized for one-factor analyses. Wald’s χ^2^ with pairwise comparisons was used in Generalized Linear Model for multifactorial analyses that did not accomplish normality or homoscedasticity. Additional individual comparisons were performed when appropriated.

For related sample analyses, two-way repeated measures ANOVA was used to assess Sex x Treatment interaction. To assess the effect of the treatments across the estrous cycle, one-way repeated measures ANOVA was performed as one factor may “overshadow” the effect of other when using multiple factors with several levels each. For repeated measures ANOVA, we used trials for FA or mean of groups of 5 trials for FE test as within-subject variable. Equality of variances and sphericity were tested. When sphericity could not be assumed, Greenhouse-Geisser statistics were used for assessing the significance and the corrected degrees of freedom were provided. ANOVA was used for the evaluation of simple main effects and when an interaction between main effects was significant. Linear regressions were calculated to understand the correlational effects between serum and amygdala hormones. The results are presented as means ± or + SEM, and statistical significance was set at P ≤ 0.05. Specific sample sizes are reported in figure legends for each dataset.

Normality and homoscedasticity tests, as well as statistics with main effects, interactions, pairwise analyses, and effect size, when appropriate, are represented in Supplementary Data 1. In Supplementary Data 2, 95% confidence intervals are represented.

All graphs presented in the figures were designed using Prism 7 (CA, USA).

### Reporting summary

Further information on research design is available in the Nature Research Reporting Summary linked to this article.

### Supplementary information

Supplementary Information

Description of Additional Supplementary Files

Supplementary Data 1

Supplementary Data 2

Reporting Summary

### Source data

Source Data

## Data Availability

All datasets obtained and analyzed in the reported study are available from the corresponding author upon reasonable request. Source data are provided with this article and deposited in the Digital Repository of Documents from the Autonomous University of Barcelona 10.5565/ddd.uab.cat/237256. Source data are provided with this paper.
