# Author Correction: Sex differences in fear memory consolidation via Tac2 signaling in mice

**DOI:** 10.1038/s41467-024-50516-5

**Published:** 2024-07-18

**Authors:** A. Florido, E. R. Velasco, C. M. Soto-Faguás, A. Gomez-Gomez, L. Perez-Caballero, P. Molina, R. Nadal, O. J. Pozo, C. A. Saura, R. Andero

**Affiliations:** 1 Institut de Neurociències, Universitat Autònoma de Barcelona, Cerdanyola del Vallès, Barcelona Spain; 2 Departament de Psicobiologia i de Metodologia de les Ciències de la Salut, Universitat Autònoma de Barcelona, Cerdanyola del Vallès, Barcelona Spain; 3 Department de Bioquímica i Biologia Molecular, Facultat de Medicina, Universitat Autònoma de Barcelona, Cerdanyola del Vallès, Barcelona Spain; 4 Centro de Investigación Biomédica en Red Enfermedades Neurodegenerativas (CIBERNED), Instituto de Salud Carlos III, Madrid, Spain; 5 Integrative Pharmacology and Systems Neuroscience Research Group, Neurosciences Research Program, IMIM (Hospital del Mar Medical Research Institute), Barcelona, Spain; 6 Unitat de Fisiologia Animal, Departament de Biologia Cel·lular, Fisiologia i Immunologia. Facultat de Biociències, Universitat Autònoma de Barcelona, Cerdanyola del Vallès, Barcelona Spain; 7 Centro de Investigación Biomédica En Red en Salud Mental (CIBERSAM), Instituto de Salud Carlos III, Madrid, Spain; 8 Unitat de Neurociència Traslacional, Parc Taulí Hospital Universitari, Institut d’Investigació i Innovació Parc Taulí (I3PT), Universitat Autònoma de Barcelona, Cerdanyola del Vallès, Barcelona Spain; 9 ICREA, Pg. Lluís Companys 23, Barcelona, Spain

Correction to: *Nature Communications* 10.1038/s41467-021-22911-9, published online 03 May 2021

The original version of this Article contained errors in the Source Data file, Supplementary Fig. 7, and figure legends for Fig. 1d and 3q.

In the Source Data tab labelled “Proestr intra-CeA,Osanetant(4b)”, the “Vehicle” and “Osanetant” groups were inadvertently switched. This has been corrected. In addition, the mouse with ID 9 was removed from experiments during procedures, but data from this mouse was included in the Source Data file and Supplementary Fig. 7. The data points from this mouse have now been removed from the Source Data File and Supplementary Fig. 7. The HTML has been updated to include a corrected version of the Supplementary information and Source Data file.

In the Source Data tab labelled “P28 mice, Osanetant (2a-b)+” there were a number of values reported incorrectly. They have been corrected in the Source Data file. The corresponding figure for this part of the Source Data did not contain these errors.

The Source Data tab labelled “Nk3R+ neurons in CeA (2d)” had an error for animal #1 (row 2 column F). The correct value, corresponding to 32 neurons in Nk3R + , is 212.7. This value has been corrected in the Source Data. The corresponding figure for this part of the Source Data did not contain this error.

The figure legend of Fig. 1d stated that experiments were “replicated three times with similar results” but these replication datasets were not included in the original version of the Article. These replication datasets have now been added to the Source Data. The legend of Fig. 1d now states “This experiment was replicated three times with similar results (see Source Data file).”

The figure legend of Fig. 3q incorrectly stated “This experiment was replicated twice with similar results” instead of “Basal levels of the estradiol experiment were replicated in Supplementary Fig. 3e with similar results”. This has been corrected.

The HTML has been updated to include a corrected version of the Source Data. The original incorrect version of the Source Data file can be found as Supplementary Information associated with this Correction.

The HTML has been updated to include a corrected version of the Supplementary information.

The errors in figure legends for Fig. 1d and 3q have been updated in the PDF and HTML versions of the article.

None of these errors change the results or their interpretation and the conclusions remain the same.

### Supplementary information

Supplementary information

### Source data

Source data

