# Supplementary material for: Sex differences in fear memory consolidation via Tac2 signaling in mice
Source: Nat Commun. 2021 May 3;12:2496. doi: 10.1038/s41467-021-22911-9 (PMC8093426; doi:10.1038/s41467-021-22911-9)
Supplement: Supplementary file 1 — Supplementary Information [file 41467_2021_22911_MOESM1_ESM.pptx]

## Slide 1
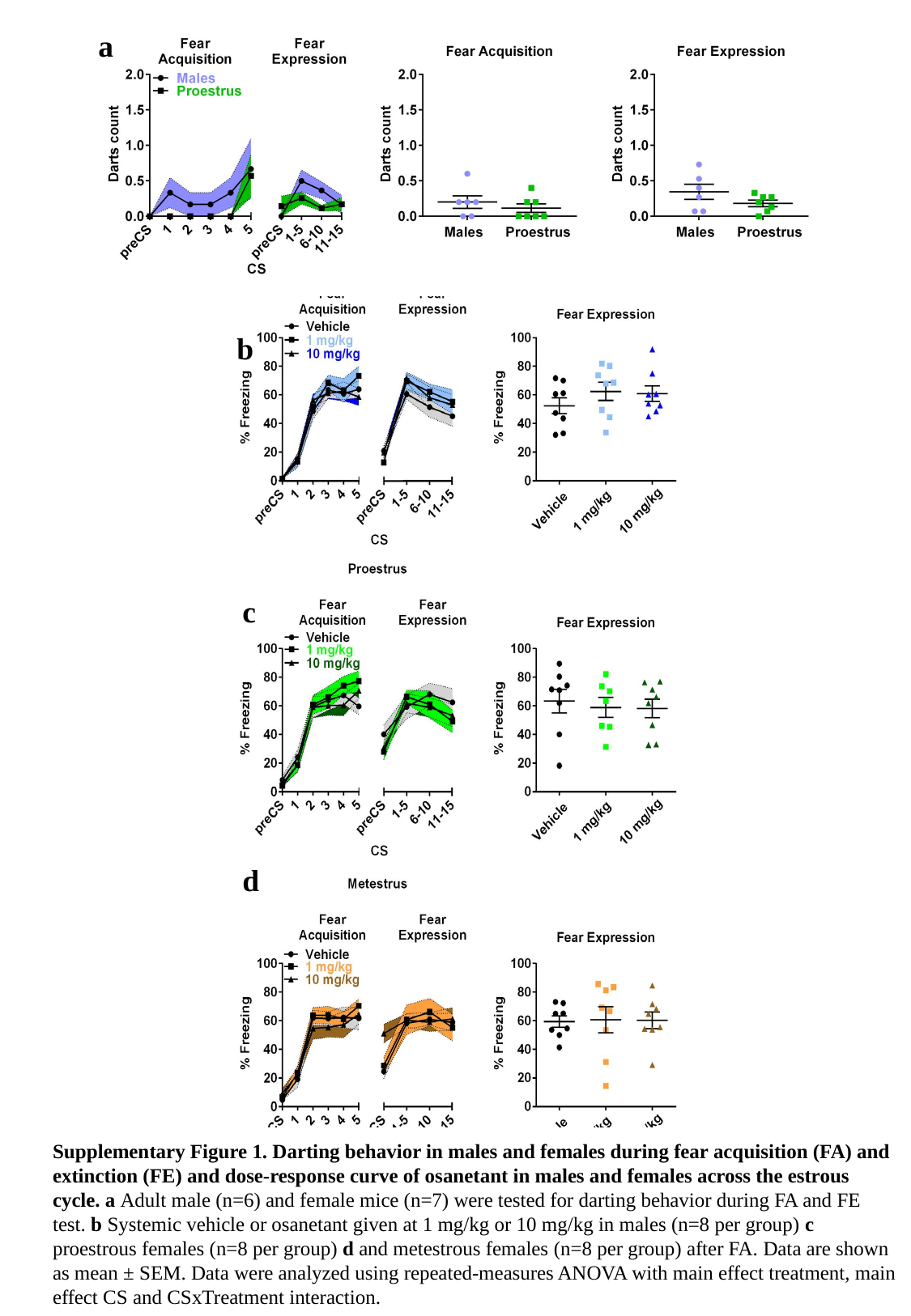

a
b
c
d
Supplementary Figure 1. Darting behavior in males and females during fear acquisition (FA) and extinction (FE) and dose-response curve of osanetant in males and females across the estrous cycle. a Adult male (n=6) and female mice (n=7) were tested for darting behavior during FA and FE test. b Systemic vehicle or osanetant given at 1 mg/kg or 10 mg/kg in males (n=8 per group) c proestrous females (n=8 per group) d and metestrous females (n=8 per group) after FA. Data are shown as mean ± SEM. Data were analyzed using repeated-measures ANOVA with main effect treatment, main effect CS and CSxTreatment interaction.

## Slide 2
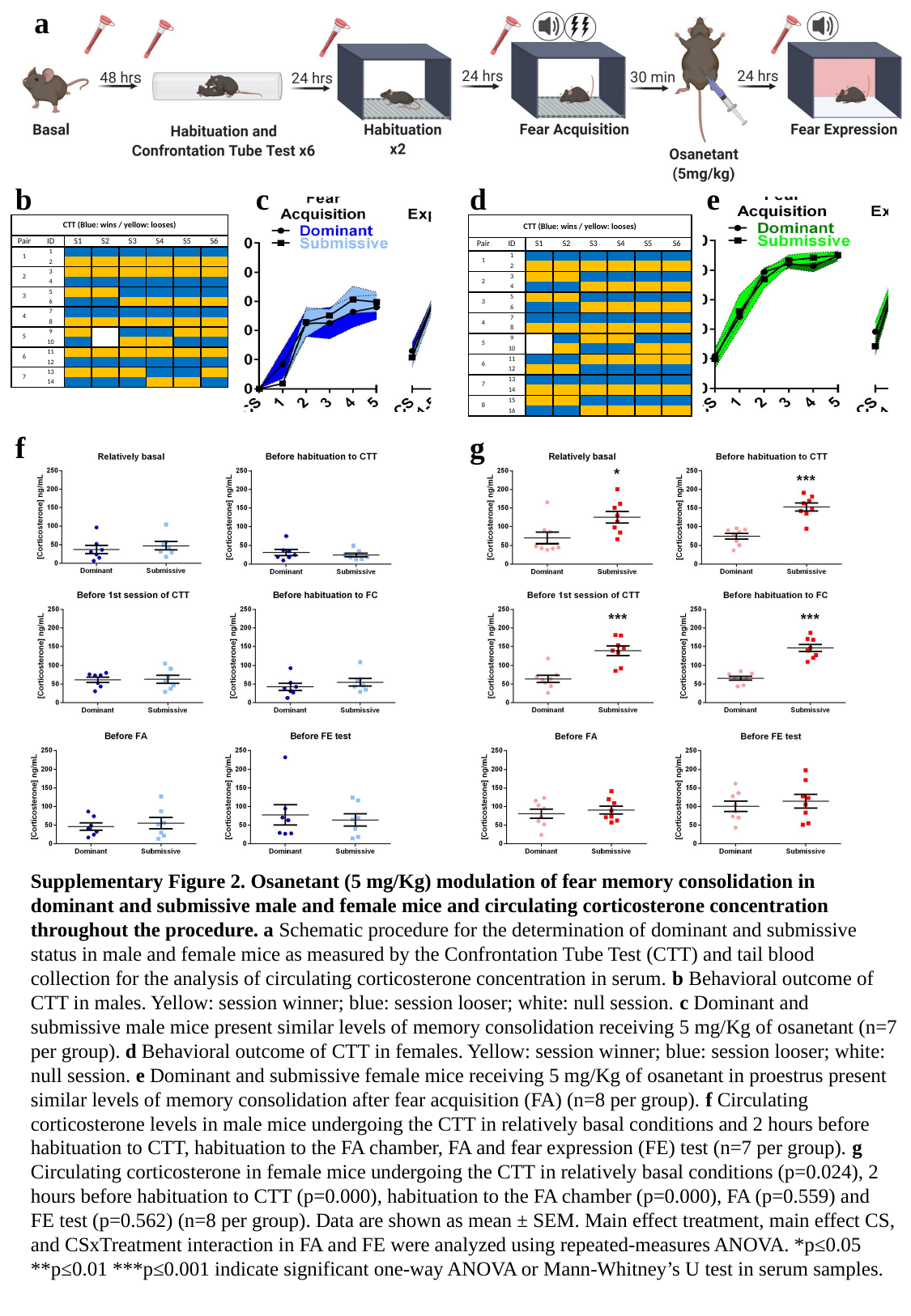

a
b
c
d
e
| CTT (Blue: wins / yellow: looses) | | | | | | | |
| --- | --- | --- | --- | --- | --- | --- | --- |
| Pair | ID | S1 | S2 | S3 | S4 | S5 | S6 |
| 1 | 1 | | | | | | |
| | 2 | | | | | | |
| 2 | 3 | | | | | | |
| | 4 | | | | | | |
| 3 | 5 | | | | | | |
| | 6 | | | | | | |
| 4 | 7 | | | | | | |
| | 8 | | | | | | |
| 5 | 9 | | | | | | |
| | 10 | | | | | | |
| 6 | 11 | | | | | | |
| | 12 | | | | | | |
| 7 | 13 | | | | | | |
| | 14 | | | | | | |
| CTT (Blue: wins / yellow: looses) | | | | | | | |
| --- | --- | --- | --- | --- | --- | --- | --- |
| Pair | ID | S1 | S2 | S3 | S4 | S5 | S6 |
| 1 | 1 | | | | | | |
| | 2 | | | | | | |
| 2 | 3 | | | | | | |
| | 4 | | | | | | |
| 3 | 5 | | | | | | |
| | 6 | | | | | | |
| 4 | 7 | | | | | | |
| | 8 | | | | | | |
| 5 | 9 | | | | | | |
| | 10 | | | | | | |
| 6 | 11 | | | | | | |
| | 12 | | | | | | |
| 7 | 13 | | | | | | |
| | 14 | | | | | | |
| 8 | 15 | | | | | | |
| | 16 | | | | | | |
f
g
Supplementary Figure 2. Osanetant (5 mg/Kg) modulation of fear memory consolidation in dominant and submissive male and female mice and circulating corticosterone concentration throughout the procedure. a Schematic procedure for the determination of dominant and submissive status in male and female mice as measured by the Confrontation Tube Test (CTT) and tail blood collection for the analysis of circulating corticosterone concentration in serum. b Behavioral outcome of CTT in males. Yellow: session winner; blue: session looser; white: null session. c Dominant and submissive male mice present similar levels of memory consolidation receiving 5 mg/Kg of osanetant (n=7 per group). d Behavioral outcome of CTT in females. Yellow: session winner; blue: session looser; white: null session. e Dominant and submissive female mice receiving 5 mg/Kg of osanetant in proestrus present similar levels of memory consolidation after fear acquisition (FA) (n=8 per group). f Circulating corticosterone levels in male mice undergoing the CTT in relatively basal conditions and 2 hours before habituation to CTT, habituation to the FA chamber, FA and fear expression (FE) test (n=7 per group). g Circulating corticosterone in female mice undergoing the CTT in relatively basal conditions (p=0.024), 2 hours before habituation to CTT (p=0.000), habituation to the FA chamber (p=0.000), FA (p=0.559) and FE test (p=0.562) (n=8 per group). Data are shown as mean ± SEM. Main effect treatment, main effect CS, and CSxTreatment interaction in FA and FE were analyzed using repeated-measures ANOVA. *p≤0.05 **p≤0.01 ***p≤0.001 indicate significant one-way ANOVA or Mann-Whitney’s U test in serum samples.

## Slide 3
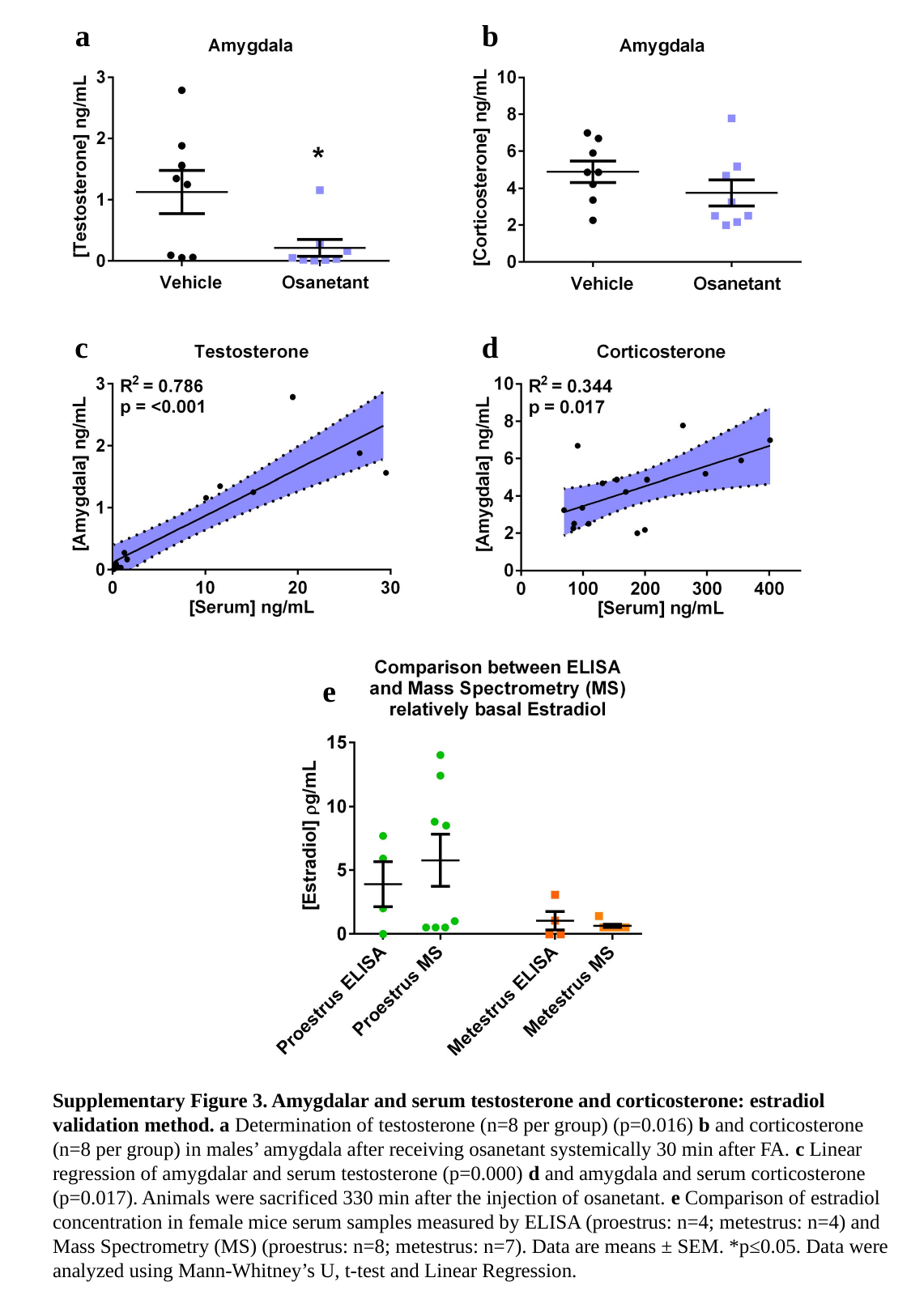

a
b
c
d
e
Supplementary Figure 3. Amygdalar and serum testosterone and corticosterone: estradiol validation method. a Determination of testosterone (n=8 per group) (p=0.016) b and corticosterone (n=8 per group) in males’ amygdala after receiving osanetant systemically 30 min after FA. c Linear regression of amygdalar and serum testosterone (p=0.000) d and amygdala and serum corticosterone (p=0.017). Animals were sacrificed 330 min after the injection of osanetant. e Comparison of estradiol concentration in female mice serum samples measured by ELISA (proestrus: n=4; metestrus: n=4) and Mass Spectrometry (MS) (proestrus: n=8; metestrus: n=7). Data are means ± SEM. *p≤0.05. Data were analyzed using Mann-Whitney’s U, t-test and Linear Regression.

## Slide 4
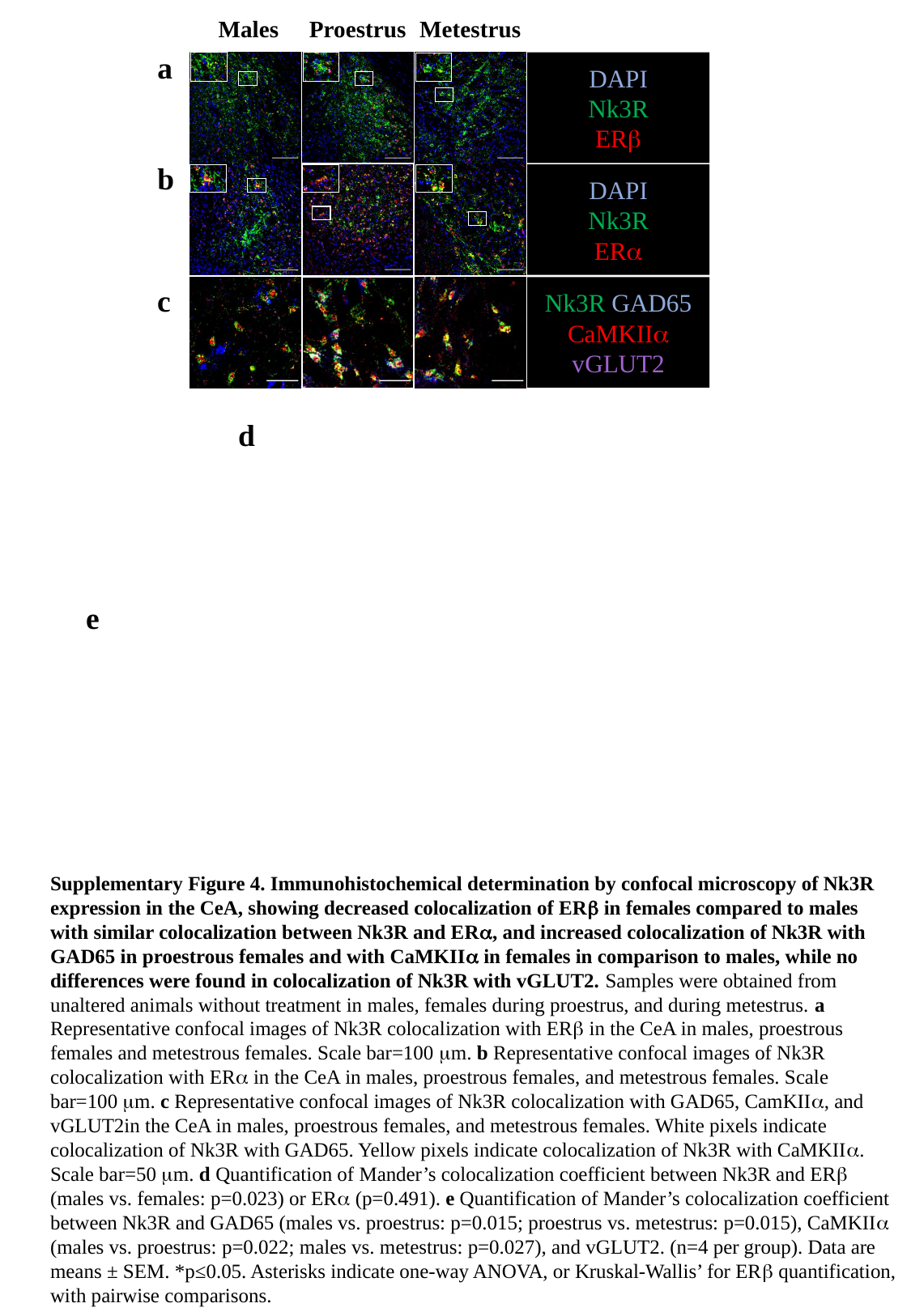

Males
Proestrus
Metestrus
a
DAPI
Nk3R
ERb
DAPI
Nk3R
ERa
Nk3R GAD65
CaMKIIa
vGLUT2
b
c
d
e
Supplementary Figure 4. Immunohistochemical determination by confocal microscopy of Nk3R expression in the CeA, showing decreased colocalization of ERb in females compared to males with similar colocalization between Nk3R and ERa, and increased colocalization of Nk3R with GAD65 in proestrous females and with CaMKIIa in females in comparison to males, while no differences were found in colocalization of Nk3R with vGLUT2. Samples were obtained from unaltered animals without treatment in males, females during proestrus, and during metestrus. a Representative confocal images of Nk3R colocalization with ERb in the CeA in males, proestrous females and metestrous females. Scale bar=100 mm. b Representative confocal images of Nk3R colocalization with ERa in the CeA in males, proestrous females, and metestrous females. Scale bar=100 mm. c Representative confocal images of Nk3R colocalization with GAD65, CamKIIa, and vGLUT2in the CeA in males, proestrous females, and metestrous females. White pixels indicate colocalization of Nk3R with GAD65. Yellow pixels indicate colocalization of Nk3R with CaMKIIa. Scale bar=50 mm. d Quantification of Mander’s colocalization coefficient between Nk3R and ERb (males vs. females: p=0.023) or ERa (p=0.491). e Quantification of Mander’s colocalization coefficient between Nk3R and GAD65 (males vs. proestrus: p=0.015; proestrus vs. metestrus: p=0.015), CaMKIIa (males vs. proestrus: p=0.022; males vs. metestrus: p=0.027), and vGLUT2. (n=4 per group). Data are means ± SEM. *p≤0.05. Asterisks indicate one-way ANOVA, or Kruskal-Wallis’ for ERb quantification, with pairwise comparisons.

## Slide 5
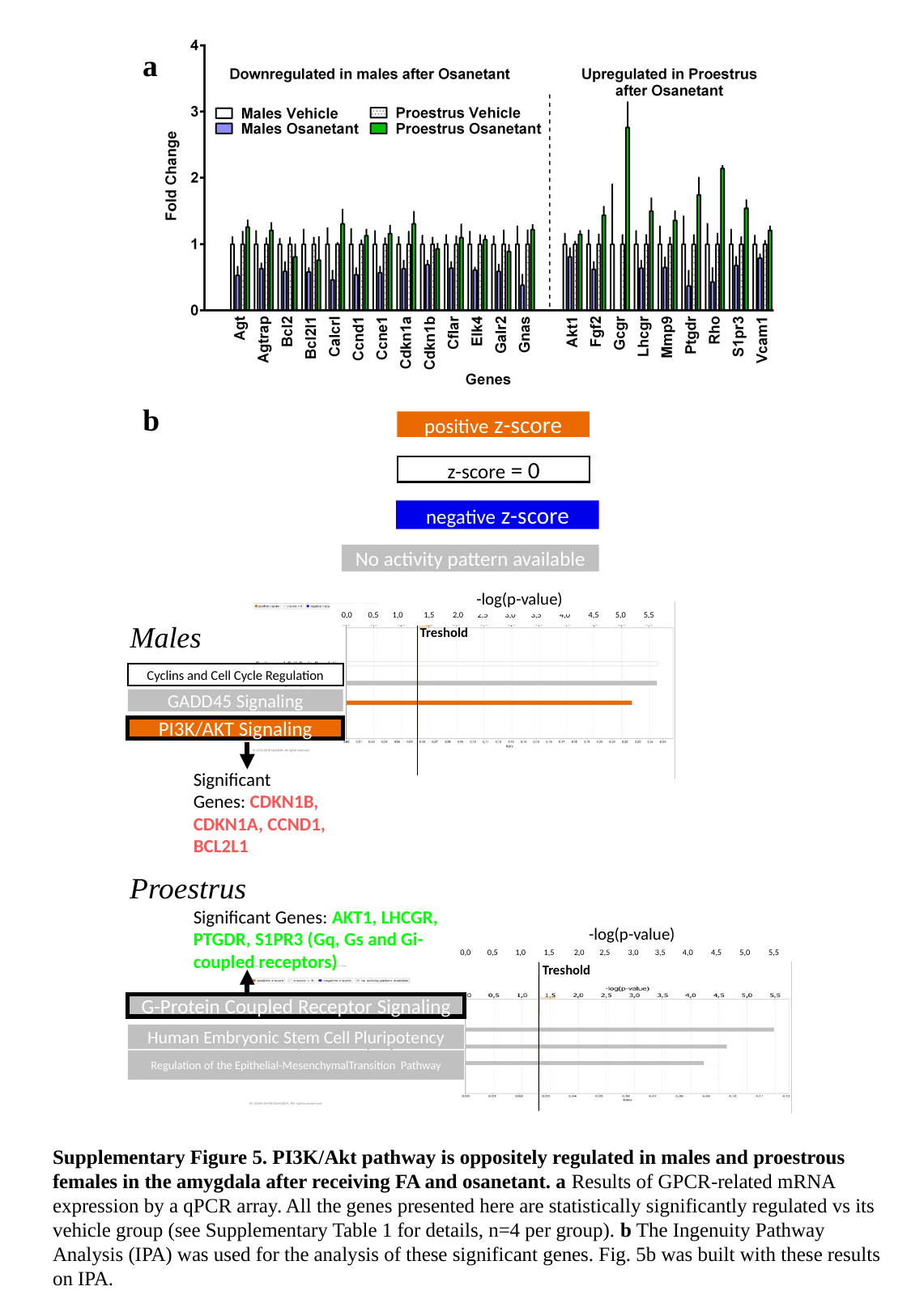

a
b
positive z-score
z-score = 0
negative z-score
No activity pattern available
-log(p-value)
0,0
0,5
1,0
1,5
2,0
2,5
3,0
3,5
4,0
4,5
5,0
5,5
Treshold
Cyclins and Cell Cycle Regulation
GADD45 Signaling
PI3K/AKT Signaling
Significant Genes: CDKN1B, CDKN1A, CCND1, BCL2L1
Significant Genes: AKT1, LHCGR, PTGDR, S1PR3 (Gq, Gs and Gi-coupled receptors)
-log(p-value)
0,0
0,5
1,0
1,5
2,0
2,5
3,0
3,5
4,0
4,5
5,0
5,5
Treshold
G-Protein Coupled Receptor Signaling
Human Embryonic Stem Cell Pluripotency
Regulation of the Epithelial-MesenchymalTransition Pathway
Supplementary Figure 5. PI3K/Akt pathway is oppositely regulated in males and proestrous females in the amygdala after receiving FA and osanetant. a Results of GPCR-related mRNA expression by a qPCR array. All the genes presented here are statistically significantly regulated vs its vehicle group (see Supplementary Table 1 for details, n=4 per group). b The Ingenuity Pathway Analysis (IPA) was used for the analysis of these significant genes. Fig. 5b was built with these results on IPA.

## Slide 6
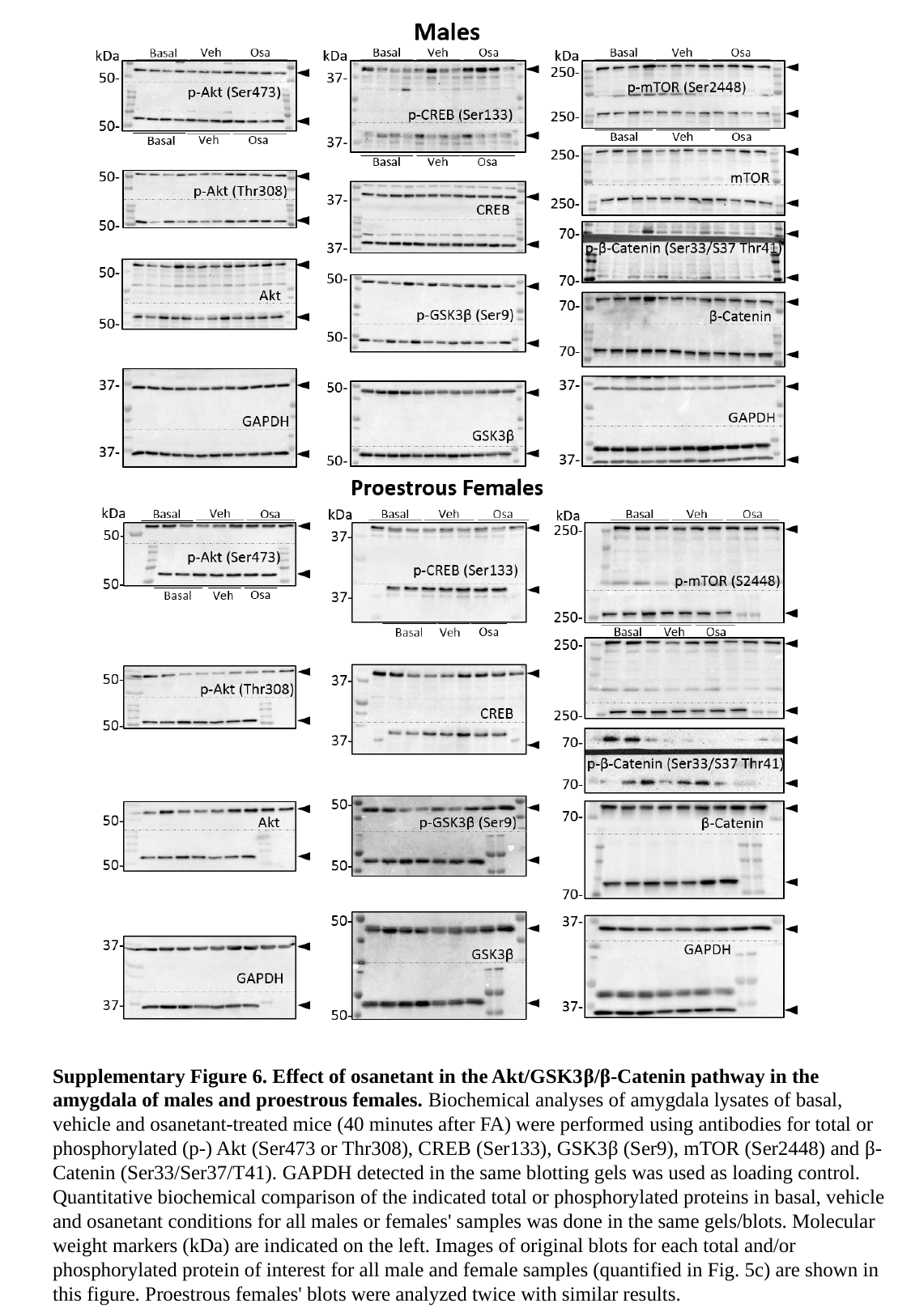

Supplementary Figure 6. Effect of osanetant in the Akt/GSK3β/β-Catenin pathway in the amygdala of males and proestrous females. Biochemical analyses of amygdala lysates of basal, vehicle and osanetant-treated mice (40 minutes after FA) were performed using antibodies for total or phosphorylated (p-) Akt (Ser473 or Thr308), CREB (Ser133), GSK3β (Ser9), mTOR (Ser2448) and β-Catenin (Ser33/Ser37/T41). GAPDH detected in the same blotting gels was used as loading control. Quantitative biochemical comparison of the indicated total or phosphorylated proteins in basal, vehicle and osanetant conditions for all males or females' samples was done in the same gels/blots. Molecular weight markers (kDa) are indicated on the left. Images of original blots for each total and/or phosphorylated protein of interest for all male and female samples (quantified in Fig. 5c) are shown in this figure. Proestrous females' blots were analyzed twice with similar results.

## Slide 7
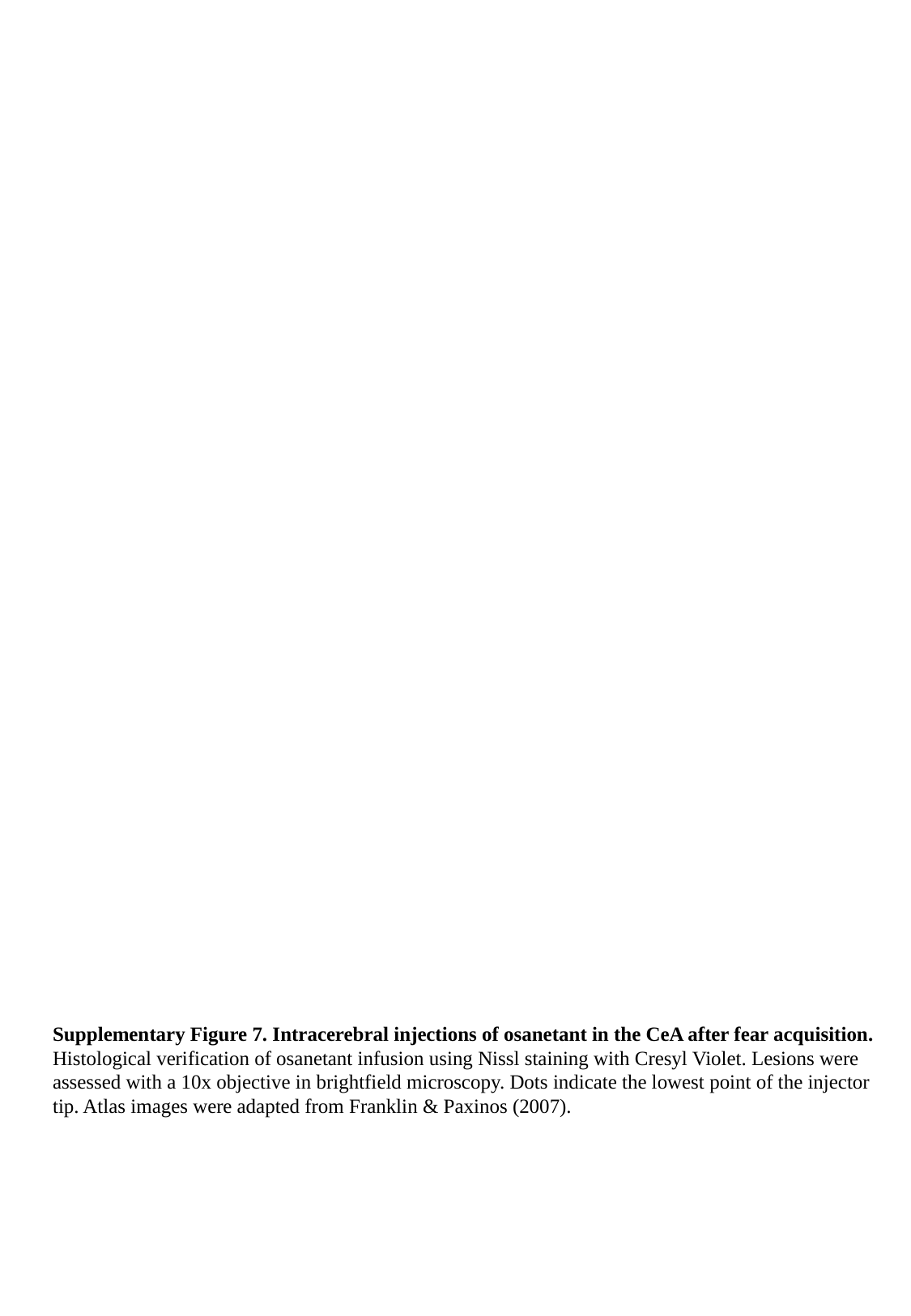

Supplementary Figure 7. Intracerebral injections of osanetant in the CeA after fear acquisition. Histological verification of osanetant infusion using Nissl staining with Cresyl Violet. Lesions were assessed with a 10x objective in brightfield microscopy. Dots indicate the lowest point of the injector tip. Atlas images were adapted from Franklin & Paxinos (2007).

## Slide 8
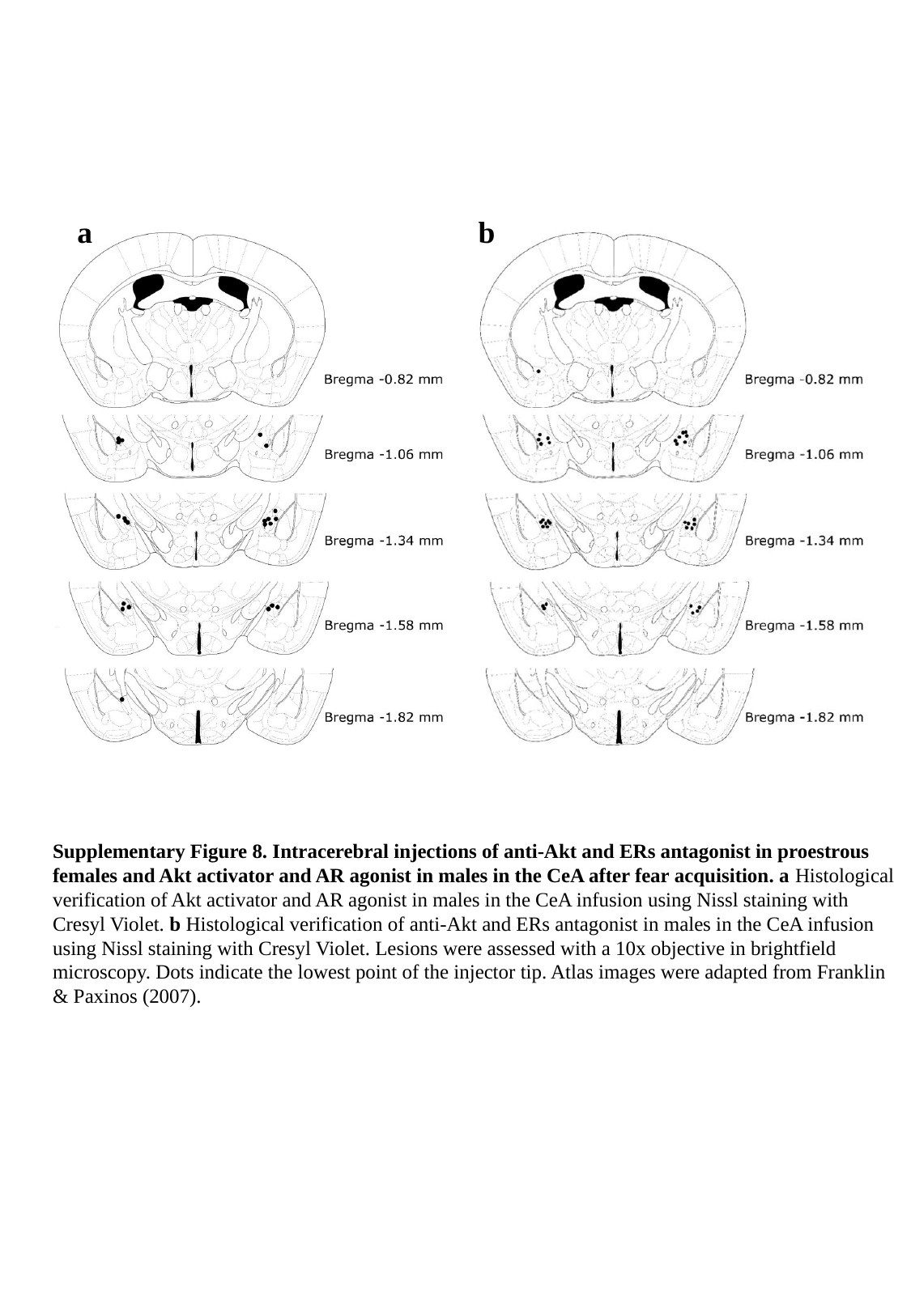

a
b
Supplementary Figure 8. Intracerebral injections of anti-Akt and ERs antagonist in proestrous females and Akt activator and AR agonist in males in the CeA after fear acquisition. a Histological verification of Akt activator and AR agonist in males in the CeA infusion using Nissl staining with Cresyl Violet. b Histological verification of anti-Akt and ERs antagonist in males in the CeA infusion using Nissl staining with Cresyl Violet. Lesions were assessed with a 10x objective in brightfield microscopy. Dots indicate the lowest point of the injector tip. Atlas images were adapted from Franklin & Paxinos (2007).

## Slide 9
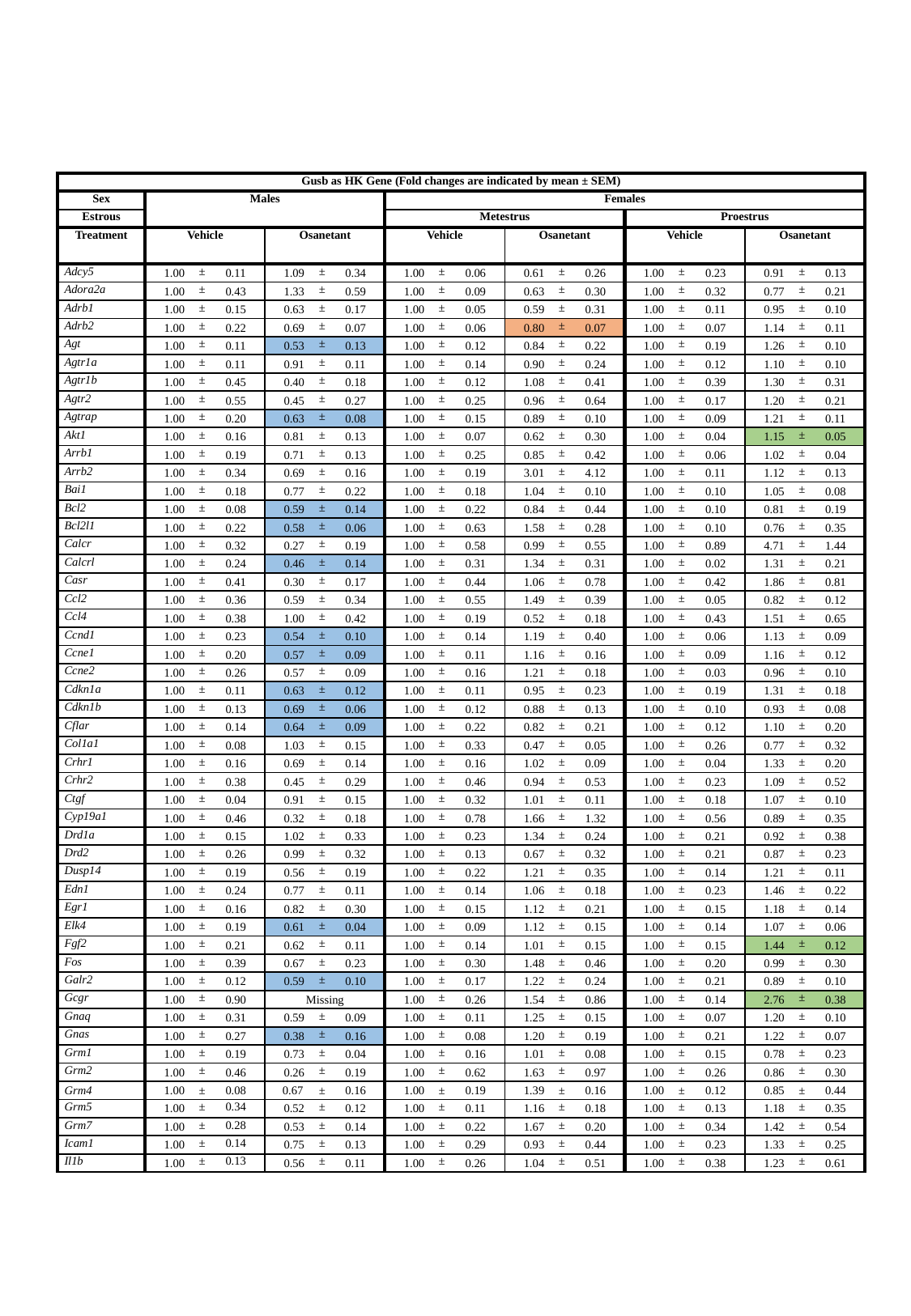

| Gusb as HK Gene (Fold changes are indicated by mean ± SEM) | | | | | | | | | | | | | | | | | | |
| --- | --- | --- | --- | --- | --- | --- | --- | --- | --- | --- | --- | --- | --- | --- | --- | --- | --- | --- |
| Sex | Males | | | | | | Females | | | | | | | | | | | |
| Estrous | | | | | | | Metestrus | | | | | | Proestrus | | | | | |
| Treatment | Vehicle | | | Osanetant | | | Vehicle | | | Osanetant | | | Vehicle | | | Osanetant | | |
| Adcy5 | 1.00 | ± | 0.11 | 1.09 | ± | 0.34 | 1.00 | ± | 0.06 | 0.61 | ± | 0.26 | 1.00 | ± | 0.23 | 0.91 | ± | 0.13 |
| Adora2a | 1.00 | ± | 0.43 | 1.33 | ± | 0.59 | 1.00 | ± | 0.09 | 0.63 | ± | 0.30 | 1.00 | ± | 0.32 | 0.77 | ± | 0.21 |
| Adrb1 | 1.00 | ± | 0.15 | 0.63 | ± | 0.17 | 1.00 | ± | 0.05 | 0.59 | ± | 0.31 | 1.00 | ± | 0.11 | 0.95 | ± | 0.10 |
| Adrb2 | 1.00 | ± | 0.22 | 0.69 | ± | 0.07 | 1.00 | ± | 0.06 | 0.80 | ± | 0.07 | 1.00 | ± | 0.07 | 1.14 | ± | 0.11 |
| Agt | 1.00 | ± | 0.11 | 0.53 | ± | 0.13 | 1.00 | ± | 0.12 | 0.84 | ± | 0.22 | 1.00 | ± | 0.19 | 1.26 | ± | 0.10 |
| Agtr1a | 1.00 | ± | 0.11 | 0.91 | ± | 0.11 | 1.00 | ± | 0.14 | 0.90 | ± | 0.24 | 1.00 | ± | 0.12 | 1.10 | ± | 0.10 |
| Agtr1b | 1.00 | ± | 0.45 | 0.40 | ± | 0.18 | 1.00 | ± | 0.12 | 1.08 | ± | 0.41 | 1.00 | ± | 0.39 | 1.30 | ± | 0.31 |
| Agtr2 | 1.00 | ± | 0.55 | 0.45 | ± | 0.27 | 1.00 | ± | 0.25 | 0.96 | ± | 0.64 | 1.00 | ± | 0.17 | 1.20 | ± | 0.21 |
| Agtrap | 1.00 | ± | 0.20 | 0.63 | ± | 0.08 | 1.00 | ± | 0.15 | 0.89 | ± | 0.10 | 1.00 | ± | 0.09 | 1.21 | ± | 0.11 |
| Akt1 | 1.00 | ± | 0.16 | 0.81 | ± | 0.13 | 1.00 | ± | 0.07 | 0.62 | ± | 0.30 | 1.00 | ± | 0.04 | 1.15 | ± | 0.05 |
| Arrb1 | 1.00 | ± | 0.19 | 0.71 | ± | 0.13 | 1.00 | ± | 0.25 | 0.85 | ± | 0.42 | 1.00 | ± | 0.06 | 1.02 | ± | 0.04 |
| Arrb2 | 1.00 | ± | 0.34 | 0.69 | ± | 0.16 | 1.00 | ± | 0.19 | 3.01 | ± | 4.12 | 1.00 | ± | 0.11 | 1.12 | ± | 0.13 |
| Bai1 | 1.00 | ± | 0.18 | 0.77 | ± | 0.22 | 1.00 | ± | 0.18 | 1.04 | ± | 0.10 | 1.00 | ± | 0.10 | 1.05 | ± | 0.08 |
| Bcl2 | 1.00 | ± | 0.08 | 0.59 | ± | 0.14 | 1.00 | ± | 0.22 | 0.84 | ± | 0.44 | 1.00 | ± | 0.10 | 0.81 | ± | 0.19 |
| Bcl2l1 | 1.00 | ± | 0.22 | 0.58 | ± | 0.06 | 1.00 | ± | 0.63 | 1.58 | ± | 0.28 | 1.00 | ± | 0.10 | 0.76 | ± | 0.35 |
| Calcr | 1.00 | ± | 0.32 | 0.27 | ± | 0.19 | 1.00 | ± | 0.58 | 0.99 | ± | 0.55 | 1.00 | ± | 0.89 | 4.71 | ± | 1.44 |
| Calcrl | 1.00 | ± | 0.24 | 0.46 | ± | 0.14 | 1.00 | ± | 0.31 | 1.34 | ± | 0.31 | 1.00 | ± | 0.02 | 1.31 | ± | 0.21 |
| Casr | 1.00 | ± | 0.41 | 0.30 | ± | 0.17 | 1.00 | ± | 0.44 | 1.06 | ± | 0.78 | 1.00 | ± | 0.42 | 1.86 | ± | 0.81 |
| Ccl2 | 1.00 | ± | 0.36 | 0.59 | ± | 0.34 | 1.00 | ± | 0.55 | 1.49 | ± | 0.39 | 1.00 | ± | 0.05 | 0.82 | ± | 0.12 |
| Ccl4 | 1.00 | ± | 0.38 | 1.00 | ± | 0.42 | 1.00 | ± | 0.19 | 0.52 | ± | 0.18 | 1.00 | ± | 0.43 | 1.51 | ± | 0.65 |
| Ccnd1 | 1.00 | ± | 0.23 | 0.54 | ± | 0.10 | 1.00 | ± | 0.14 | 1.19 | ± | 0.40 | 1.00 | ± | 0.06 | 1.13 | ± | 0.09 |
| Ccne1 | 1.00 | ± | 0.20 | 0.57 | ± | 0.09 | 1.00 | ± | 0.11 | 1.16 | ± | 0.16 | 1.00 | ± | 0.09 | 1.16 | ± | 0.12 |
| Ccne2 | 1.00 | ± | 0.26 | 0.57 | ± | 0.09 | 1.00 | ± | 0.16 | 1.21 | ± | 0.18 | 1.00 | ± | 0.03 | 0.96 | ± | 0.10 |
| Cdkn1a | 1.00 | ± | 0.11 | 0.63 | ± | 0.12 | 1.00 | ± | 0.11 | 0.95 | ± | 0.23 | 1.00 | ± | 0.19 | 1.31 | ± | 0.18 |
| Cdkn1b | 1.00 | ± | 0.13 | 0.69 | ± | 0.06 | 1.00 | ± | 0.12 | 0.88 | ± | 0.13 | 1.00 | ± | 0.10 | 0.93 | ± | 0.08 |
| Cflar | 1.00 | ± | 0.14 | 0.64 | ± | 0.09 | 1.00 | ± | 0.22 | 0.82 | ± | 0.21 | 1.00 | ± | 0.12 | 1.10 | ± | 0.20 |
| Col1a1 | 1.00 | ± | 0.08 | 1.03 | ± | 0.15 | 1.00 | ± | 0.33 | 0.47 | ± | 0.05 | 1.00 | ± | 0.26 | 0.77 | ± | 0.32 |
| Crhr1 | 1.00 | ± | 0.16 | 0.69 | ± | 0.14 | 1.00 | ± | 0.16 | 1.02 | ± | 0.09 | 1.00 | ± | 0.04 | 1.33 | ± | 0.20 |
| Crhr2 | 1.00 | ± | 0.38 | 0.45 | ± | 0.29 | 1.00 | ± | 0.46 | 0.94 | ± | 0.53 | 1.00 | ± | 0.23 | 1.09 | ± | 0.52 |
| Ctgf | 1.00 | ± | 0.04 | 0.91 | ± | 0.15 | 1.00 | ± | 0.32 | 1.01 | ± | 0.11 | 1.00 | ± | 0.18 | 1.07 | ± | 0.10 |
| Cyp19a1 | 1.00 | ± | 0.46 | 0.32 | ± | 0.18 | 1.00 | ± | 0.78 | 1.66 | ± | 1.32 | 1.00 | ± | 0.56 | 0.89 | ± | 0.35 |
| Drd1a | 1.00 | ± | 0.15 | 1.02 | ± | 0.33 | 1.00 | ± | 0.23 | 1.34 | ± | 0.24 | 1.00 | ± | 0.21 | 0.92 | ± | 0.38 |
| Drd2 | 1.00 | ± | 0.26 | 0.99 | ± | 0.32 | 1.00 | ± | 0.13 | 0.67 | ± | 0.32 | 1.00 | ± | 0.21 | 0.87 | ± | 0.23 |
| Dusp14 | 1.00 | ± | 0.19 | 0.56 | ± | 0.19 | 1.00 | ± | 0.22 | 1.21 | ± | 0.35 | 1.00 | ± | 0.14 | 1.21 | ± | 0.11 |
| Edn1 | 1.00 | ± | 0.24 | 0.77 | ± | 0.11 | 1.00 | ± | 0.14 | 1.06 | ± | 0.18 | 1.00 | ± | 0.23 | 1.46 | ± | 0.22 |
| Egr1 | 1.00 | ± | 0.16 | 0.82 | ± | 0.30 | 1.00 | ± | 0.15 | 1.12 | ± | 0.21 | 1.00 | ± | 0.15 | 1.18 | ± | 0.14 |
| Elk4 | 1.00 | ± | 0.19 | 0.61 | ± | 0.04 | 1.00 | ± | 0.09 | 1.12 | ± | 0.15 | 1.00 | ± | 0.14 | 1.07 | ± | 0.06 |
| Fgf2 | 1.00 | ± | 0.21 | 0.62 | ± | 0.11 | 1.00 | ± | 0.14 | 1.01 | ± | 0.15 | 1.00 | ± | 0.15 | 1.44 | ± | 0.12 |
| Fos | 1.00 | ± | 0.39 | 0.67 | ± | 0.23 | 1.00 | ± | 0.30 | 1.48 | ± | 0.46 | 1.00 | ± | 0.20 | 0.99 | ± | 0.30 |
| Galr2 | 1.00 | ± | 0.12 | 0.59 | ± | 0.10 | 1.00 | ± | 0.17 | 1.22 | ± | 0.24 | 1.00 | ± | 0.21 | 0.89 | ± | 0.10 |
| Gcgr | 1.00 | ± | 0.90 | Missing | | | 1.00 | ± | 0.26 | 1.54 | ± | 0.86 | 1.00 | ± | 0.14 | 2.76 | ± | 0.38 |
| Gnaq | 1.00 | ± | 0.31 | 0.59 | ± | 0.09 | 1.00 | ± | 0.11 | 1.25 | ± | 0.15 | 1.00 | ± | 0.07 | 1.20 | ± | 0.10 |
| Gnas | 1.00 | ± | 0.27 | 0.38 | ± | 0.16 | 1.00 | ± | 0.08 | 1.20 | ± | 0.19 | 1.00 | ± | 0.21 | 1.22 | ± | 0.07 |
| Grm1 | 1.00 | ± | 0.19 | 0.73 | ± | 0.04 | 1.00 | ± | 0.16 | 1.01 | ± | 0.08 | 1.00 | ± | 0.15 | 0.78 | ± | 0.23 |
| Grm2 | 1.00 | ± | 0.46 | 0.26 | ± | 0.19 | 1.00 | ± | 0.62 | 1.63 | ± | 0.97 | 1.00 | ± | 0.26 | 0.86 | ± | 0.30 |
| Grm4 | 1.00 | ± | 0.08 | 0.67 | ± | 0.16 | 1.00 | ± | 0.19 | 1.39 | ± | 0.16 | 1.00 | ± | 0.12 | 0.85 | ± | 0.44 |
| Grm5 | 1.00 | ± | 0.34 | 0.52 | ± | 0.12 | 1.00 | ± | 0.11 | 1.16 | ± | 0.18 | 1.00 | ± | 0.13 | 1.18 | ± | 0.35 |
| Grm7 | 1.00 | ± | 0.28 | 0.53 | ± | 0.14 | 1.00 | ± | 0.22 | 1.67 | ± | 0.20 | 1.00 | ± | 0.34 | 1.42 | ± | 0.54 |
| Icam1 | 1.00 | ± | 0.14 | 0.75 | ± | 0.13 | 1.00 | ± | 0.29 | 0.93 | ± | 0.44 | 1.00 | ± | 0.23 | 1.33 | ± | 0.25 |
| Il1b | 1.00 | ± | 0.13 | 0.56 | ± | 0.11 | 1.00 | ± | 0.26 | 1.04 | ± | 0.51 | 1.00 | ± | 0.38 | 1.23 | ± | 0.61 |

## Slide 10
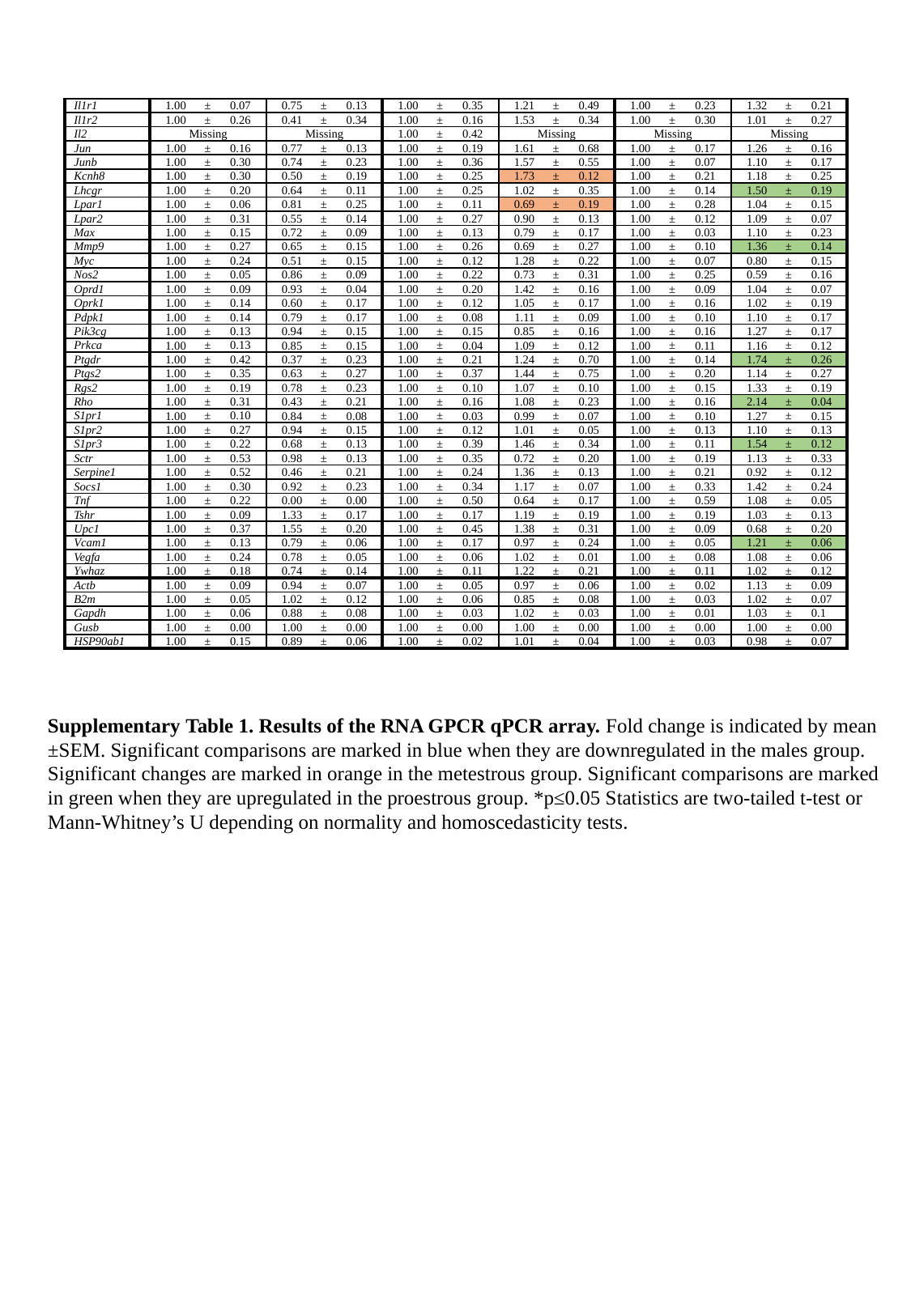

| Il1r1 | 1.00 | ± | 0.07 | 0.75 | ± | 0.13 | 1.00 | ± | 0.35 | 1.21 | ± | 0.49 | 1.00 | ± | 0.23 | 1.32 | ± | 0.21 |
| --- | --- | --- | --- | --- | --- | --- | --- | --- | --- | --- | --- | --- | --- | --- | --- | --- | --- | --- |
| Il1r2 | 1.00 | ± | 0.26 | 0.41 | ± | 0.34 | 1.00 | ± | 0.16 | 1.53 | ± | 0.34 | 1.00 | ± | 0.30 | 1.01 | ± | 0.27 |
| Il2 | Missing | | | Missing | | | 1.00 | ± | 0.42 | Missing | | | Missing | | | Missing | | |
| Jun | 1.00 | ± | 0.16 | 0.77 | ± | 0.13 | 1.00 | ± | 0.19 | 1.61 | ± | 0.68 | 1.00 | ± | 0.17 | 1.26 | ± | 0.16 |
| Junb | 1.00 | ± | 0.30 | 0.74 | ± | 0.23 | 1.00 | ± | 0.36 | 1.57 | ± | 0.55 | 1.00 | ± | 0.07 | 1.10 | ± | 0.17 |
| Kcnh8 | 1.00 | ± | 0.30 | 0.50 | ± | 0.19 | 1.00 | ± | 0.25 | 1.73 | ± | 0.12 | 1.00 | ± | 0.21 | 1.18 | ± | 0.25 |
| Lhcgr | 1.00 | ± | 0.20 | 0.64 | ± | 0.11 | 1.00 | ± | 0.25 | 1.02 | ± | 0.35 | 1.00 | ± | 0.14 | 1.50 | ± | 0.19 |
| Lpar1 | 1.00 | ± | 0.06 | 0.81 | ± | 0.25 | 1.00 | ± | 0.11 | 0.69 | ± | 0.19 | 1.00 | ± | 0.28 | 1.04 | ± | 0.15 |
| Lpar2 | 1.00 | ± | 0.31 | 0.55 | ± | 0.14 | 1.00 | ± | 0.27 | 0.90 | ± | 0.13 | 1.00 | ± | 0.12 | 1.09 | ± | 0.07 |
| Max | 1.00 | ± | 0.15 | 0.72 | ± | 0.09 | 1.00 | ± | 0.13 | 0.79 | ± | 0.17 | 1.00 | ± | 0.03 | 1.10 | ± | 0.23 |
| Mmp9 | 1.00 | ± | 0.27 | 0.65 | ± | 0.15 | 1.00 | ± | 0.26 | 0.69 | ± | 0.27 | 1.00 | ± | 0.10 | 1.36 | ± | 0.14 |
| Myc | 1.00 | ± | 0.24 | 0.51 | ± | 0.15 | 1.00 | ± | 0.12 | 1.28 | ± | 0.22 | 1.00 | ± | 0.07 | 0.80 | ± | 0.15 |
| Nos2 | 1.00 | ± | 0.05 | 0.86 | ± | 0.09 | 1.00 | ± | 0.22 | 0.73 | ± | 0.31 | 1.00 | ± | 0.25 | 0.59 | ± | 0.16 |
| Oprd1 | 1.00 | ± | 0.09 | 0.93 | ± | 0.04 | 1.00 | ± | 0.20 | 1.42 | ± | 0.16 | 1.00 | ± | 0.09 | 1.04 | ± | 0.07 |
| Oprk1 | 1.00 | ± | 0.14 | 0.60 | ± | 0.17 | 1.00 | ± | 0.12 | 1.05 | ± | 0.17 | 1.00 | ± | 0.16 | 1.02 | ± | 0.19 |
| Pdpk1 | 1.00 | ± | 0.14 | 0.79 | ± | 0.17 | 1.00 | ± | 0.08 | 1.11 | ± | 0.09 | 1.00 | ± | 0.10 | 1.10 | ± | 0.17 |
| Pik3cg | 1.00 | ± | 0.13 | 0.94 | ± | 0.15 | 1.00 | ± | 0.15 | 0.85 | ± | 0.16 | 1.00 | ± | 0.16 | 1.27 | ± | 0.17 |
| Prkca | 1.00 | ± | 0.13 | 0.85 | ± | 0.15 | 1.00 | ± | 0.04 | 1.09 | ± | 0.12 | 1.00 | ± | 0.11 | 1.16 | ± | 0.12 |
| Ptgdr | 1.00 | ± | 0.42 | 0.37 | ± | 0.23 | 1.00 | ± | 0.21 | 1.24 | ± | 0.70 | 1.00 | ± | 0.14 | 1.74 | ± | 0.26 |
| Ptgs2 | 1.00 | ± | 0.35 | 0.63 | ± | 0.27 | 1.00 | ± | 0.37 | 1.44 | ± | 0.75 | 1.00 | ± | 0.20 | 1.14 | ± | 0.27 |
| Rgs2 | 1.00 | ± | 0.19 | 0.78 | ± | 0.23 | 1.00 | ± | 0.10 | 1.07 | ± | 0.10 | 1.00 | ± | 0.15 | 1.33 | ± | 0.19 |
| Rho | 1.00 | ± | 0.31 | 0.43 | ± | 0.21 | 1.00 | ± | 0.16 | 1.08 | ± | 0.23 | 1.00 | ± | 0.16 | 2.14 | ± | 0.04 |
| S1pr1 | 1.00 | ± | 0.10 | 0.84 | ± | 0.08 | 1.00 | ± | 0.03 | 0.99 | ± | 0.07 | 1.00 | ± | 0.10 | 1.27 | ± | 0.15 |
| S1pr2 | 1.00 | ± | 0.27 | 0.94 | ± | 0.15 | 1.00 | ± | 0.12 | 1.01 | ± | 0.05 | 1.00 | ± | 0.13 | 1.10 | ± | 0.13 |
| S1pr3 | 1.00 | ± | 0.22 | 0.68 | ± | 0.13 | 1.00 | ± | 0.39 | 1.46 | ± | 0.34 | 1.00 | ± | 0.11 | 1.54 | ± | 0.12 |
| Sctr | 1.00 | ± | 0.53 | 0.98 | ± | 0.13 | 1.00 | ± | 0.35 | 0.72 | ± | 0.20 | 1.00 | ± | 0.19 | 1.13 | ± | 0.33 |
| Serpine1 | 1.00 | ± | 0.52 | 0.46 | ± | 0.21 | 1.00 | ± | 0.24 | 1.36 | ± | 0.13 | 1.00 | ± | 0.21 | 0.92 | ± | 0.12 |
| Socs1 | 1.00 | ± | 0.30 | 0.92 | ± | 0.23 | 1.00 | ± | 0.34 | 1.17 | ± | 0.07 | 1.00 | ± | 0.33 | 1.42 | ± | 0.24 |
| Tnf | 1.00 | ± | 0.22 | 0.00 | ± | 0.00 | 1.00 | ± | 0.50 | 0.64 | ± | 0.17 | 1.00 | ± | 0.59 | 1.08 | ± | 0.05 |
| Tshr | 1.00 | ± | 0.09 | 1.33 | ± | 0.17 | 1.00 | ± | 0.17 | 1.19 | ± | 0.19 | 1.00 | ± | 0.19 | 1.03 | ± | 0.13 |
| Upc1 | 1.00 | ± | 0.37 | 1.55 | ± | 0.20 | 1.00 | ± | 0.45 | 1.38 | ± | 0.31 | 1.00 | ± | 0.09 | 0.68 | ± | 0.20 |
| Vcam1 | 1.00 | ± | 0.13 | 0.79 | ± | 0.06 | 1.00 | ± | 0.17 | 0.97 | ± | 0.24 | 1.00 | ± | 0.05 | 1.21 | ± | 0.06 |
| Vegfa | 1.00 | ± | 0.24 | 0.78 | ± | 0.05 | 1.00 | ± | 0.06 | 1.02 | ± | 0.01 | 1.00 | ± | 0.08 | 1.08 | ± | 0.06 |
| Ywhaz | 1.00 | ± | 0.18 | 0.74 | ± | 0.14 | 1.00 | ± | 0.11 | 1.22 | ± | 0.21 | 1.00 | ± | 0.11 | 1.02 | ± | 0.12 |
| Actb | 1.00 | ± | 0.09 | 0.94 | ± | 0.07 | 1.00 | ± | 0.05 | 0.97 | ± | 0.06 | 1.00 | ± | 0.02 | 1.13 | ± | 0.09 |
| B2m | 1.00 | ± | 0.05 | 1.02 | ± | 0.12 | 1.00 | ± | 0.06 | 0.85 | ± | 0.08 | 1.00 | ± | 0.03 | 1.02 | ± | 0.07 |
| Gapdh | 1.00 | ± | 0.06 | 0.88 | ± | 0.08 | 1.00 | ± | 0.03 | 1.02 | ± | 0.03 | 1.00 | ± | 0.01 | 1.03 | ± | 0.1 |
| Gusb | 1.00 | ± | 0.00 | 1.00 | ± | 0.00 | 1.00 | ± | 0.00 | 1.00 | ± | 0.00 | 1.00 | ± | 0.00 | 1.00 | ± | 0.00 |
| HSP90ab1 | 1.00 | ± | 0.15 | 0.89 | ± | 0.06 | 1.00 | ± | 0.02 | 1.01 | ± | 0.04 | 1.00 | ± | 0.03 | 0.98 | ± | 0.07 |
Supplementary Table 1. Results of the RNA GPCR qPCR array. Fold change is indicated by mean ±SEM. Significant comparisons are marked in blue when they are downregulated in the males group. Significant changes are marked in orange in the metestrous group. Significant comparisons are marked in green when they are upregulated in the proestrous group. *p≤0.05 Statistics are two-tailed t-test or Mann-Whitney’s U depending on normality and homoscedasticity tests.

## Slide 11
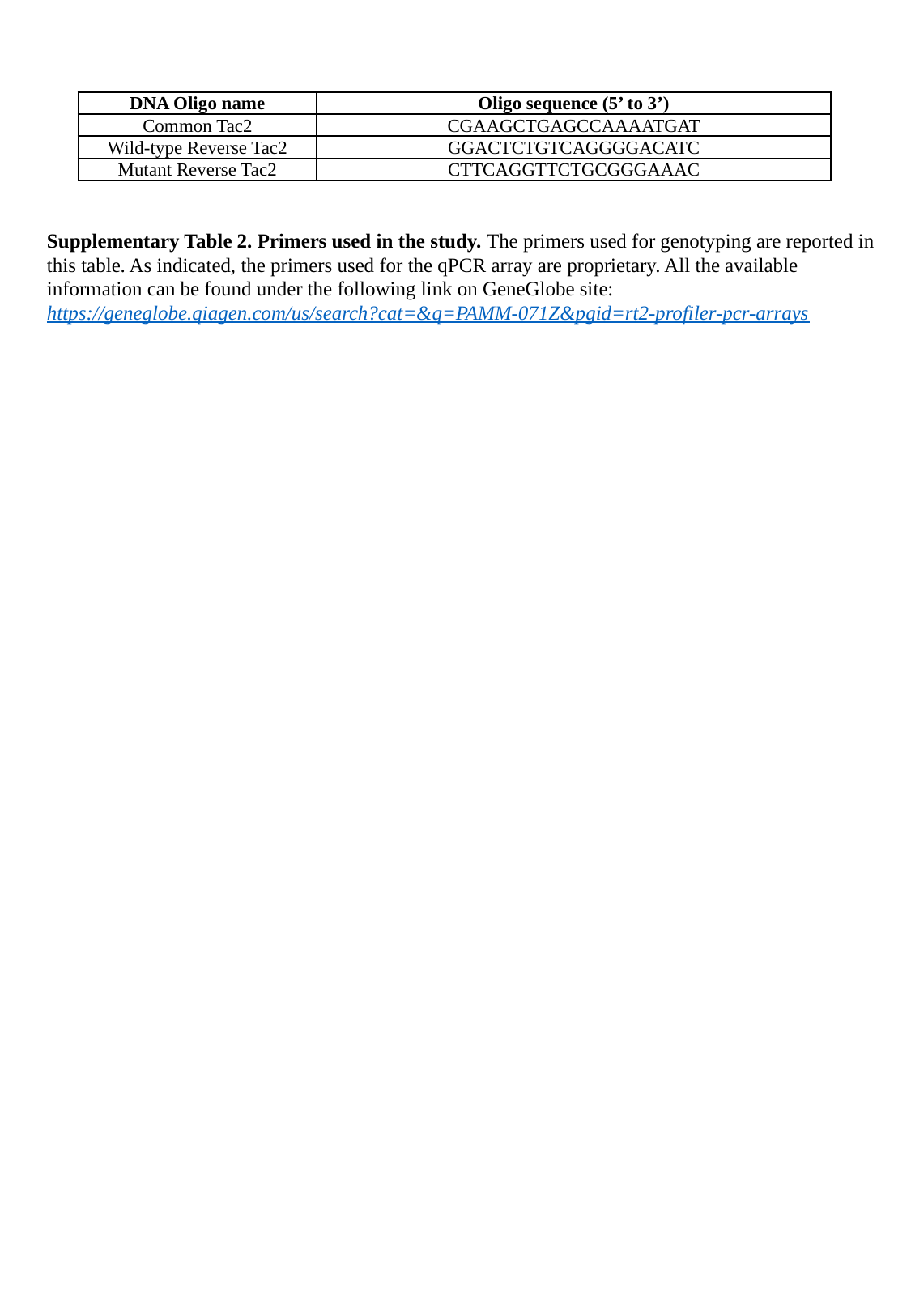

| DNA Oligo name | Oligo sequence (5’ to 3’) |
| --- | --- |
| Common Tac2 | CGAAGCTGAGCCAAAATGAT |
| Wild-type Reverse Tac2 | GGACTCTGTCAGGGGACATC |
| Mutant Reverse Tac2 | CTTCAGGTTCTGCGGGAAAC |
Supplementary Table 2. Primers used in the study. The primers used for genotyping are reported in this table. As indicated, the primers used for the qPCR array are proprietary. All the available information can be found under the following link on GeneGlobe site: https://geneglobe.qiagen.com/us/search?cat=&q=PAMM-071Z&pgid=rt2-profiler-pcr-arrays
