# Supplementary material for: Sex differences in fear memory consolidation via Tac2 signaling in mice
Source: Nat Commun. 2021 May 3;12:2496. doi: 10.1038/s41467-021-22911-9 (PMC8093426; doi:10.1038/s41467-021-22911-9)
Supplement: Supplementary file 2 — Description of Additional Supplementary Files [file 41467_2021_22911_MOESM2_ESM.docx]

**Description of Additional Supplementary Files**

File Name: **Supplementary Data 1.**

Description: **Statistical analyses of all experiments with 95% confidence intervals.** For related sample analyses, Box’s M test was used to assess homoscedasticity and Mauchly’s W to assess sphericity. For independent samples comparisons, Kolmogorov-Smirnov and Shapiro-Wilk were employed to assess normality and Levene’s test to assess homoscedasticity, and One-way or Two-way ANOVA from the General Linear Model (GLM) were used to assess statistically significant differences. Two-tailed non-parametric tests were used for unifactorial analyses that did not reach the GLM criteria.

For multifactorial analyses, Wald’s χ^2^ from the Generalized Linear Model (GzLM) was used to assess significance. Additional two-tailed pairwise comparisons when required.

File Name: **Supplementary Data 2.**

Description: **95% Confidence interval for each dataset.**
